# Genetic selection for temperament traits in dairy and beef cattle

**DOI:** 10.3389/fgene.2014.00368

**Published:** 2014-10-21

**Authors:** Marie J. Haskell, Geoff Simm, Simon P. Turner

**Affiliations:** Animal and Veterinary Sciences Group, Scotland's Rural CollegeEdinburgh, UK

**Keywords:** temperament, animal welfare, genetic variation, animal personality, genetic correlation

## Abstract

Animal temperament can be defined as a response to environmental or social stimuli. There are a number of temperament traits in cattle that contribute to their welfare, including their response to handling or milking, response to challenge such as human approach or intervention at calving, and response to conspecifics. In a number of these areas, the genetic basis of the trait has been studied. Heritabilities have been estimated and in some cases quantitative trait loci (QTL) have been identified. The variation is sometimes considerable and moderate heritabilities have been found for the major handling temperament traits, making them amenable to selection. Studies have also investigated the correlations between temperament and other traits, such as productivity and meat quality. Despite this, there are relatively few examples of temperament traits being used in selection programmes. Most often, animals are screened for aggression or excessive fear during handling or milking, with extreme animals being culled, or EBVs for temperament are estimated, but these traits are not commonly included routinely in selection indices, despite there being economic, welfare and human safety drivers for their. There may be a number of constraints and barriers. For some traits and breeds, there may be difficulties in collecting behavioral data on sufficiently large populations of animals to estimate genetic parameters. Most selection indices require estimates of economic values, and it is often difficult to assign an economic value to a temperament trait. The effects of selection primarily for productivity traits on temperament and welfare are discussed. Future opportunities include automated data collection methods and the wider use of genomic information in selection.

## Introduction

Genetic improvement, including selection between breeds, crossing and within-breed selection, is widely used in farm livestock and has led to dramatic changes in performance in dairy and beef cattle over the last 50 years or so (e.g., Simm, 1998). Historically, most emphasis has been on traits that are most directly associated with profitability, and most easily measured, such as milk yield or body weight. However, selection between or within breeds for a broader set of traits, including health and “fitness” traits, is becoming more widespread as producers realize that productivity can only be maintained or improved with a more holistic view of animal performance. Reproduction, longevity and health traits are used in a number of breeding programmes for dairy and beef cattle, and there is growing interest in behavioral traits associated with animal welfare and ease of management. Temperament traits such as fearfulness or aggressiveness are important to consider as they affect how the animal responds to the husbandry and handling conditions on the farm and during procedures like transport. The aim of this review is to determine what progress has been made in the steps in the chain from trait definition through to the use of these traits in selection, including the recent opportunity for genomic selection. We also review the research that has investigated associations between temperament traits and productivity, health and reproductive traits to determine whether selection for these traits may be altering temperament indirectly.

## What is temperament?

Farmers and others involved with the keeping of cattle and other livestock are well aware that there are differences between individual animals in their behavioral response to alarming or challenging situations. Furthermore, individuals are often consistent in the way they respond when the challenge is repeated. In cattle, the magnitude of response, and the difference between animals are of most importance to humans in situations that involve human interaction, such as where animals are handled, moved or milked. Some animals are calm and docile, while others are distressed and struggle to escape. Animals may also show consistency in their response in other situations, such as response to a new-born calf, and aggression or affiliation toward herd-mates.

This observed consistency of response within the animal, and the variation shown between individual animals or groups of animals, has historically been given a number of different labels, depending on whether the user is from a psychological, farm livestock or behavioral ecology background. In human psychology, it is known as personality, while in behavioral ecology the term “behavioral syndrome” is used to describe differences in clustering of traits between animal populations. In animal husbandry settings, the term “temperament” is largely used. In cattle, temperament is often described as an animal's response to handling or forced movement by humans (Tulloh, 1961; Burrow, 1997). This definition appears to have come from the terminology that farmers use to describe the way their cattle behave during handling (e.g., Hassall, 1974, a paper from a beef producer). It is also similar to the term “disposition” used in North America (Beef Improvement Federation Guidelines, 2010). This human-focussed definition of temperament has been used broadly across the cattle sector, particularly in beef cattle. A number of authors have used the term “temperament” with a situation “specifier” to describe the context (e.g., Brown, 1974 uses the term “maternal protective temperament”). Thus the term “handling temperament” can be used to differentiate the response from other contexts. The use of terms such as “maternal temperament” and “aggressive temperament” or simply a descriptor term such as “aggressiveness” and “sociability” are found in studies that consider consistency in the animal's response in contexts other than handling (Brown, 1974; Kilgour and Dalton, 1984; Reale et al., 2007; Gutierrez-Gil et al., 2008; Gibbons et al., 2009a, 2010).

## Why is temperament important?

The temperament traits that have received most attention are generally those that have adverse production, welfare or human safety consequences. The foremost of these is handling temperament, and the impacts of poor temperament on farm management efficiency and animals has been a key driver for many studies (e.g., Burrow, 1997; Barrozo et al., 2012). A beef animal that responds to confinement in a chute, weigh crush or handling race by struggling violently and trying to escape is at a higher risk of injury to itself, human handlers and other animals than an animal that responds calmly (Voisinet et al., 1997a). This type of animal is also more likely to make the process of handling a group of animals for weighing or drafting much slower and less efficient. A number of studies have shown that handling temperament is also linked to growth, feeding efficiency and meat quality in beef cattle. Understanding the extent of this association has driven a great deal of research that will be discussed below. For dairy cattle, a calm response to the milking procedure is important both to maximize the efficiency of the milking process and to minimize the residual milk volume. Docility in dairy cattle at milking and during handling is a trait that has been under selection informally and formally for generations, so extreme responses are rare. However, the problems created by an animal that is not easy to handle and milk mean that “dairy temperament” (which is measured as strength of response to the milking procedure) has been investigated and is still part of many dairy breeding programmes worldwide (Interbull:www.interbull.org/ib/geforms).

There are other temperament traits that have received less attention in the literature, but are important from an animal welfare or human safety standpoint. Maternal aggressiveness, where a dam shows defensive aggressiveness toward any human or animal attempting to interfere with her calf, is a trait that clearly had evolutionary advantages for wild animals, and still does in some extensive production environments. However, when this aggression is directed at stockworkers or members of the public entering grazing fields, it clearly becomes much more problematic (Turner et al., 2013). Other traits that are important for welfare include resource-based aggression, where an animal shows aggression toward another when in competition for a resource such as feed or water, and social motivation or sociability, which is the willingness to be in close proximity to group-mates.

## Trait definition and measurement

**Figure 1:**
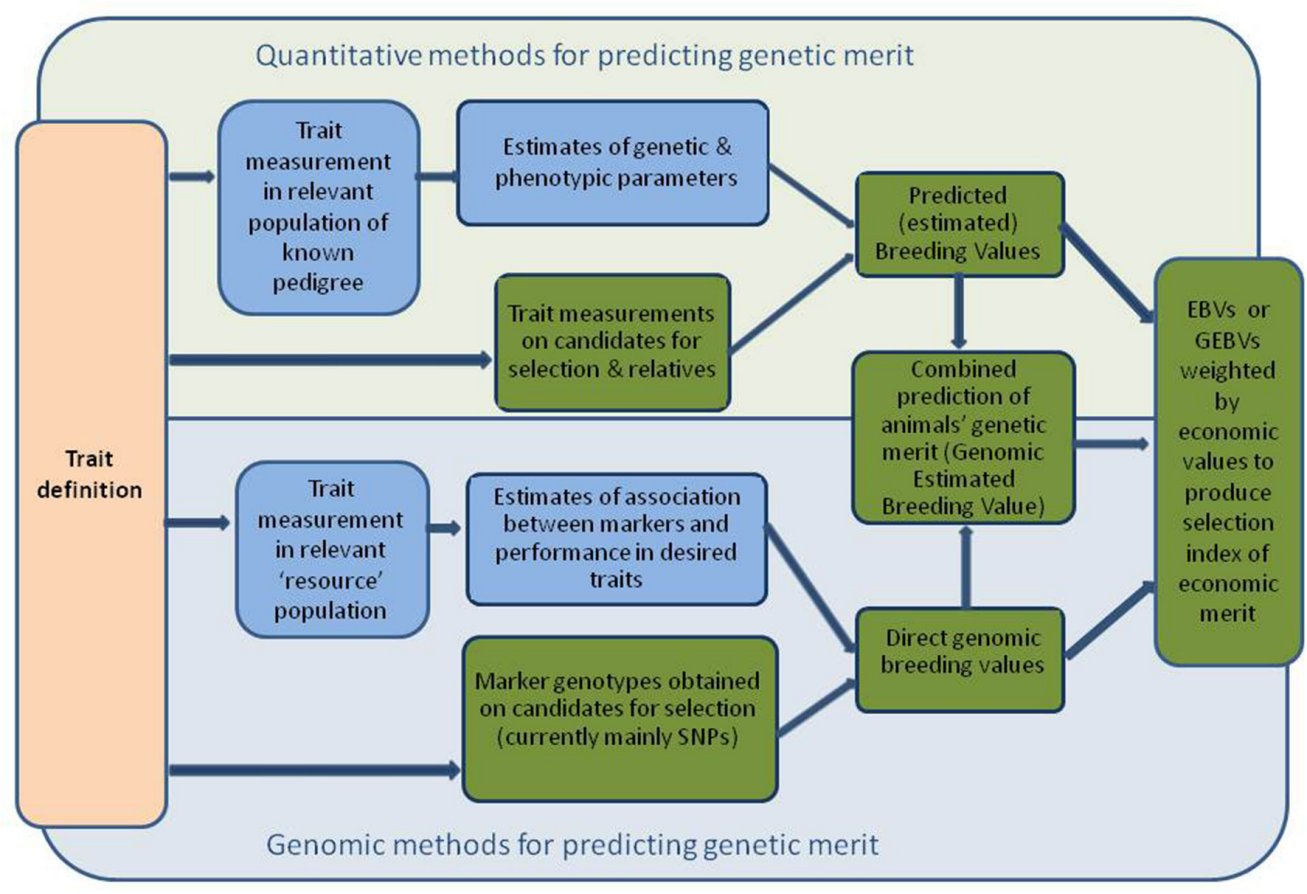
**Diagram showing sequence of process from trait definition to estimation of breeding values or genomic breeding values, and ultimately to GEBVs**. Blue fill indicates that these are processes required at the initiation of a breeding programme and are updated periodically. Green fill indicates routine processes on each cohort of candidate animals for selection.

Given the potential adverse effects of excitable temperament on human safety and handling efficiency, the use of selective breeding to improve temperament is important. A number of steps must be taken to enable selection to take place (Figure 1). Firstly, the trait (in this case a behavior or response) must be defined, which typically includes a definition of the context in which it is important. The next step is to devise a measurement system so that the trait can be assessed in a rapid, quantifiable and reliable way by non-scientists, and then to validate it against other measures of the trait if possible, so that the chosen measure accurately characterizes the response. This measure can then be used in a number of ways. It can be used as a “screening” tool, such as when individual animals with poor scores for a temperament trait are culled or not considered for breeding, or the measure can be used as part of a genetic improvement programme. This section will investigate the progress with regards to trait definition and measurement.

### Handling—beef cattle

Fearful or excitable responses may be expressed by animals in many novel or challenging contexts, such as during interactions with other animals or when entering a new field or pen, but it is largely during handling that this characteristic becomes a problem. A fearful response to handling manifests itself in a variety of ways. Animals may struggle, show agitated movements, attempt to escape, vocalize, show increased respiration rates, defaecate, show changes in their ear, head and tail positions and facial expressions and be more or less motivated to move away from the handling area or handler. The challenge is to find a scale or measure that adequately represents these varied responses. In beef cattle, there are some very well established assessments: flight speed or flight time, chute (known as a crush in Australasia/Europe) score and the docility score. These have sometimes been grouped into restrained and non-restrained categories (Burrow, 1997). Restrained tests are primarily those assessing the response to restraint in a handling chute, confinement in a pen or raceway, or alternatively measuring the response to that confinement by assessing the flight time or speed to move away from the place of confinement. Unrestrained tests are those in which the animal is not confined, but the animal's response to being approached, moved or handled is scored. These unrestrained tests are also characterized by more directly measuring the response to proximity to a human, whereas the restrained tests may measure the response to physical restraint as well as the proximity to humans. The main tests are described below.

#### Flight speed/time

The flight speed or flight time assessment was originally used by Burrow et al. (1988) and has been widely used by groups in Australia and elsewhere. The assessment typically takes place as part of a routine weighing or handling procedure, where the animal is held in a handling system, such as a race or chute. Once the procedure is complete, the animal is released from the chute. The time it takes to cover a set distance along a raceway is calculated. This distance is typically short to capture the immediate response to release (e.g., 1.7 m: Burrow and Dillon, 1997; Cafe et al., 2011b; 1.83 m: Curley et al., 2006a). This can be presented as a velocity (e.g., “exit velocity”; Curley et al., 2006a) or as a “flight time” for a set distance (e.g., Fell et al., 1999).

#### Chute test

The chute test assesses the strength of response to confinement, whilst the animal is inside the chute. It is made on a categorical scale (typically 1–5), with qualitative or descriptive definitions given to states of increasing agitation, from no response, docile or calm through to vigorous, wild or violent response (e.g., Tulloh, 1961; Hearnshaw et al., 1979; Grandin, 1993). Similar categorical scoring systems have been used to quantify the response to confinement in handling races or pens (e.g., Fordyce et al., 1985).

#### Docility test

The main type of unrestrained test is a “docility test” in which the animal is separated from its group mates and moved to another pen. After a short period, the handler tries to drive the animal to a corner of this pen and hold it there for a predetermined period of time without physical aids. The responses to all parts of the test are integrated into a single score, but scores for the component parts can also be analyzed (Boivin et al., 1994; Le Neindre et al., 1995).

Some authors also score response to human approach in a pen on a categorical scale (e.g., King et al., 2006). Similar to this is an assessment of flight distance, which is the distance at which an animal starts to move away from an approaching human (Fisher et al., 2001). This is similar to the approach/avoidance distance assessments used in dairy cattle (Waiblinger et al., 2003; Gibbons et al., 2009b).

Animal responses to each of these measures of temperament have been shown to be repeatable over time (e.g., Hearnshaw and Morris, 1984; Grandin, 1993; Burrow and Dillon, 1997; Gibbons et al., 2009b; Turner et al., 2011). It is of interest to understand whether these different tests measure the same underlying trait. A number of studies have found a significant relationship between the measures. In beef cattle, flight speed and chute test score have been found to be significantly moderately correlated (e.g., Fell et al., 1999; Olmos and Turner, 2008; Hoppe et al., 2010; Cafe et al., 2011b) and positive correlations between chute score and flight speed, and chute score and docility have also been shown (Turner et al., 2011). Grignard et al. (2001) found a significant relationship between the docility test and the chute test in Limousin cattle, with and without a human present in front of the chute. Additionally, Curley et al. (2006b) found a moderate relationship between chute scores and response to confinement in a pen. These relationships are not found universally; others have reported weaker correlations (Burrow and Corbet, 2000), or variations in strength of the correlations between breeds (Cafe et al., 2011b). Overall, this would suggest that these tests are assessing similar if not identical underlying traits.

### Handling and milking—dairy cattle

Typically, milking temperament is seen as the response to the whole milking procedure, and is mostly scored by the farmer or milking staff. A categorical scale based on descriptive definitions of different levels of response to the milking and handling procedures are often used, with scores from 1–5 or 1–9 typically representing poor to good milking temperament. Temperament scores are often combined with other assessments such as milking speed to derive a “workability” trait. Milking temperament data are collated by herd improvement or milk recording organizations in many countries (www.interbull.org). A number of researchers have used more objective assessments such as an assessment of the number of steps, kicks or flinches the cow makes in response to the milking procedure (e.g., Willis, 1983; Breuer et al., 2000). In experimental situations, human approach or flight distance tests have been used with dairy cattle (e.g., Waiblinger et al., 2003; Gibbons et al., 2009b), and shown to have good within-animal repeatability. The tests involve scoring the response of the animal as the experimenter moves toward her. Gibbons et al. (2011) found that approach distance was related to flight speed, but not chute score, in dairy heifers.

### Other traits

Other temperament traits such as sociability, intra-specific aggression and response to novelty and social separation have been assessed in beef and dairy cattle, and maternal behavior in beef cattle. Maternal behavior, or maternal aggressiveness is a human safety as well as a calf survival issue, particularly in farming systems in which humans come in close contact with cows and calves (Turner et al., 2013). Improvement of animal welfare is the main driver for assessing many of the other traits, as well as the desire to understand the relationship between the specific handling tests and the wider personality of the animal (Kilgour et al., 2006). It is thought that animals that are not excessively fearful of novel objects or isolation from other animals will cope better with modern intensive or semi-intensive farming systems than more reactive animals (Kilgour et al., 2006; Gibbons et al., 2009b). Similarly, it has been hypothesized that an animal with high social motivation will integrate and cope better with group housing than low sociability animals, and that animals showing low aggression will suffer less stress and have less negative impact on other animals (Gibbons et al., 2009a, 2010). Methods to quantify these characteristics have involved assessing the response of animals to novel objects, social isolation or to a competitive situation. A number of studies have shown moderate to high repeatabilities of scores for individual animals, indicating that they can be classed as temperament traits (e.g., novelty: Kilgour et al., 2006; Gibbons et al., 2009b, 2010; aggression: Gibbons et al., 2009a; MacKay et al., 2013).

In terms of trait definition, it would appear that there are some very good definitions for a number of temperament traits, particularly for beef handling and dairy cow milking temperament. These traits have established measurement protocols and measurement scales. There are other traits that have received less attention, but which show good repeatability.

## Genetic variation between and within breeds

Once a trait has been defined and a reliable measurement system created, the degree of genetic variation within and between breeds must be determined, if genetic improvement is to be made. Genetic variation can be exploited in one of three ways currently—selection between breeds (or breed substitution—replacing one breed with another, superior breed), crossbreeding (crossing different breeds to create animals with intermediate performance to the parent breeds, or to produce animals with attributes of both parental breeds, or to exploit heterosis or “hybrid vigor”—the boost in performance often seen in crosses, over and above that expected from the mean performance of the parent breeds), or selection within breeds. Whether crossbreeding leads to significant heterosis effects on temperament traits such as handling ease has not been studied, but warrants investigation (Burrow, 1997). A fourth option, direct genetic modification, is also available, but this is largely confined to experimental use rather than commercial practice at the moment. This may change as techniques such as “gene editing” used in human gene therapy begin to be applied to allow targeted changes in livestock (Lilico et al., 2013).

### Breed differences

The choice of breed or strain by producers is influenced by temperament, but choice is often based on subjective information. Differences in performance of breeds managed in the same environment provide more objective evidence that a trait is under genetic control. Substitution of one breed by another is a rapid way to effect genetic change. Information on the differences between breeds and their crosses is also a prerequisite for the design of optimal crossbreeding schemes. Stark differences in handling ease between the relatively docile *Bos taurus* and relatively flighty *Bos indicus* cattle are well known (Hearnshaw et al., 1979; Becker and Lobato, 1997; Voisinet et al., 1997a; Buchenauer, 1999; Burrow, 2001). Large differences between individual breeds of *Bos taurus* cattle have also been demonstrated, although individual reports often conflict (e.g., Hearnshaw and Morris, 1984; Gauly et al., 2001; Boissy et al., 2005; Hoppe et al., 2010). There are also reported differences between the dairy breeds in their milking temperament (Sewalem et al., 2010). In many cases, these reported differences are most likely to be due to differences in the way in which cattle from the different breeds were raised and their level of exposure to humans. However, in those studies in which the rearing environment was standardized, breed differences have still been found, indicating that the response of cattle to handling by humans is, at least at the level of the breed, under some genetic control. Other than the distinction between *Bos indicus* and *Bos taurus*, studies in which different breeds have been reared and handled together in a standardized manner are not numerous enough to allow a “league table” of breed temperament to be created at present.

### Genetic variation within breeds

Having identified the optimal breeds or crosses for a given production system, there are opportunities for further genetic improvement via selection of the best parents within the chosen breed, or within each of the breeds making up the chosen crossbreed. Objective within-breed selection usually requires knowledge of the traits affecting profitability (breeding goal traits) and their relative economic values, potential proxy traits on which to base selection (selection criteria) if breeding goal traits can not be measured directly (e.g., if they are expressed late in life, can only be measured *post-mortem* or are time-consuming and costly to measure), and estimates of the genetic and phenotypic variances and covariances among these traits.

Typically, estimates of heritability (the ratio of additive genetic variation to total phenotypic variation) are required to establish the degree to which the traits of interest are under genetic control, and hence the scope for changing them by selection (the variation in the trait concerned is also important here). Accurate estimates of heritability require measures of the trait of interest, as well as pedigree information, on many animals. Heritability estimates alone are sufficient to produce simple (univariate) predicted or estimated breeding values (EBVs) which are predictions of the genetic merit of candidates for individual traits of interest. Most modern breeding programmes use more sophisticated statistical techniques (based on “best linear unbiased prediction”) to produce multivariate EBVs (a suite of EBVs for traits of interest, that takes into account relationships among animals, and associations among traits). This requires estimates of phenotypic and genetic variances and covariances among all traits (these are also required to derive regressions or correlations usually used to quantify associations among traits). Often multivariate EBVs are weighted and combined in a selection index, producing a single score identifying animals with the highest predicted genetic merit for overall economic performance. This requires estimates of the economic value of all traits that contribute to the overall breeding goal. Figure 1 illustrates the steps involved in prediction of conventional breeding values.

#### Heritability estimates for temperament traits

The extent of current knowledge on the heritability of temperament is reviewed in the section below. A large number of studies have estimated heritability for the three major handling traits in beef cattle and also for milking temperament in dairy cattle (Tables 1–4). A smaller number of studies have also investigated the heritability of other temperament traits (Table 5). There are also a number of previous reviews on the genetics of behavior (Burrow, 1997; Buchenauer, 1999; Wiener, in press).

**Table 1:** **Heritability estimates for the chute test in beef cattle**.

**References**	**Breed and sample size**	**Age at test**	**Confinement context and score**	**Heritability ± *SE***
Shrode and Hammack, 1971	Hereford (58)	Yearling	Squeeze chute (1–**5**)	0.40 ± 0.30
	Angus (114)			
Sato, 1981	Japanese Black/Shorthorn (*n* = 200)	Calves to adult	Weigh scale (1–**4**)	0.45 *P* < 0.05
Fordyce et al., 1982	Bos indicus cross and Hereford-Shorthorn cross (*n* ~ 957)	9–10 or 21–22 months	Movement in crush (1–**7**)	0.25 ± 0.20
			Audible respiration in a crush (1–**4**)	0.20 ± 0.16
			Movement in race (1–**7**)	0.17 ± 0.21
			Audible respiration in a race (1–**4**)	0.57 ± 0.22
			Movement in a headbail (1–**7**)	0.67 ± 0.26
Hearnshaw and Morris, 1984	Bos taurus	8 months	Chute (0–**5**)	0.03 ± 0.28
	Bos indicus-sired			0.46 ± 0.37
Fordyce et al., 1996	Bos indicus crosses (*n* = 485; *n* = 312 for 12 months)	Weaning	Handling/confinement in a race (1–**13.5**)	0.14 ± 0.11
		12 months		0.12 ± 0.11
		24 months		0.08 ± 0.10
Burrow and Corbet, 2000	Bos indicus cross (*n* = 851)	12–36 months	Weigh crate (1–**5**)	0.30
Schmutz et al., 2001	Bos Taurus (130)	6–12 months	Weight scale “Habituation” (difference between two repeats of test)	0.36
				0.46
Beckman et al., 2007	Limousin (21,932)	Weaning	Chute (1–**6**)	0.34 ± 0.01
Benhajali et al., 2009	Limousin (1,271)	8 months	Chute score (1–**5)**	0.18 ± 0.07–0.09
			No. of rush movements (1–**6**)	0.23 ± 0.07–0.09
			Total no. movements (1–**6**)	0.29 ± 0.07–0.09
Kadel et al., 2006	2358 Bos indicus (Brahman, Santa Gertrudis, Belmont Red)	8 months	Chute score (1–**15**)	0.19 ± 0.02
		19 months		0.15 ± 0.03
Benhajali et al., 2010	Limousin (2,141)	5 and 7 months	Weigh crate	
			TW: no. of movements	5 months: 0.14 ± 0.09
				7 months: 0.31 ± 0.10
			CTW: categorical score of TW	5 months: 0.16 ± 0.08
				7 months: 0.29 ± 0.10
			RW: no. rush movements	5 months: 0.11 ± 0.07
				7 months: 0.28 ± 0.09
			CRW: categorical score of RW	5 months: 0.11 ± 0.07
				7 months: 0.23 ± 0.09
Hoppe et al., 2010	German Angus (706)	5–11 months	Chute score (1–**5**)	0.15 ± 0.06
	Charolais (556)			0.17 ± 0.07
	Hereford (697)			0.33 ± 0.10
	Limousin (424)			0.11 ± 0.08
	German Simmental (667)			0.18 ± 0.07
Barrozo et al., 2012	Nellore (37,692)	Long yearlings (12+ months)	Corralled and human presence (1–**4**)	0.18 ± 0.02

The context refers to the location or situation in which the confinement or restraint was recorded. Sample size is shown in parentheses with breed. The scale used to measure the temperament trait is shown with the most excitable/nervous score shown in bold.

**Table 2:** Heritability estimates for flight speed (m/s) and flight time (s^*^100).

**References**	**Breed and sample size**	**Age at test**	**Measure**	**Heritability ± *SE***
Burrow et al., 1988	Bos indicus derived (561)	Weaning (42 sires)	Flight speed (m/s)	0.54 ± 0.16
		18 m (38 sires)		0.26 ± 0.13
Burrow and Corbet, 2000	Zebu-derived *n* = 851 (Duckponds popn)	12 months 2–4×	Flight speed score (rating: slow to fast)	0.08
			Flight speed	0.35
	Zebu-derived *N* = 1277 (Belmont popn)	Weaning	Flight speed score	0.39
		12 months		0.33
		18 months		0.29
Burrow, 2001	Zebu-derived (Belmont Red) (1871)	Weaning, 12 and 18 months	Flight speed	0.44 direct
				0.05 maternal effects
Johnston et al., 2003	Tropically adapted (Brahman, Belmont Red and Santa Gertrudis) (7622)	Post-weaning	Flight time	0.31 ± 0.03–0.06
Prayaga and Henshall, 2005	European and Zebu breeds (2555)	*N* = ~2555	Flight time	0.20 ± 0.03 (direct)
Kadel et al., 2006	Bos indicus: Brahman, Santa Gertrudis and Belmont Red (3594)	8 months	Flight time	0.30 ± 0.02
		19 months		0.34 ± 0.03
Nkrumah et al., 2007	Bos taurus: Angus/Charolais/beef hybrid (302)	8 months	Flight speed	0.49 ± 0.18
Rolfe et al., 2011	Bos taurus (Hereford, Angus others) (1141)	Finishing phase	Flight speed	0.34 ± 0.11
Hoppe et al., 2010	German Angus (706)	5–11 months	Flight speed score (1–4: walk to jump out of chute)	0.20 ± 0.08
	Charolais (556)			0.25 ± 0.10
	Hereford (697)			0.36 ± 0.06
	Limousin (424)			0.11 ± 0.07
	German Simmental (667)			0.28 ± 0.07

High flight speeds and low flight times indicate animals with excitable temperaments.

***Handling—beef cattle***. For the handling temperament traits, there is a wide range of heritabilities, from low to moderate, indicating that some genetic progress can be made in selective breeding programs for these traits (See Tables 1–3 for heritability estimates for beef cattle for chute tests, flight speed and docility tests, respectively). However, variation among estimates is sometimes high. The unweighted mean and range of heritabilties (irrespective of the models used) for the three traits are in the same range [chute scores/response to restraint: 0.24 (0.03–0.67); flight speed: 0.36 (0.05–0.7), and docility: 0.26 (0.0–0.61)]. Burrow (1997) concluded that despite the different types of methodologies involved, the estimates of heritability were similar (0.36 for non-restrained and 0.23 for restrained tests). Some of the difference in estimates may be explained by sampling bias alone. However, it is also likely that the variability in estimates for temperament traits given the same name is partly due to differences in measuring protocols or recording method, or to breed differences. Heritability estimates do vary between breeds, and are generally higher for *Bos indicus* breeds and crosses than for *Bos taurus* breeds. *Bos taurus* breeds of British and continental European origin have been bred for longer, in less extensive conditions, with a higher level of human contact than *Bos indicus* breeds. This history may have produced animals that are genetically less predisposed to fear humans and restraint, and which show less genetic variation in response to handling. There appears to be little maternal genetic effect on measures of offspring temperament (maternal heritabilities for flight time: 0–0.03; Prayaga and Henshall, 2005; chute test score: 0.01 to 0.05 for the different models used; Beckman et al., 2007).

**Table 3:** **Heritability estimates for docility and flight distance**.

**References**	**Breed and sample size**	**Age at test**	**Measure**	**Heritability ± *SE***
Le Neindre et al., 1995	Limousin heifers (904)	10 months	Docility score	0.22
			Docility criterion (categorical score of docility test)	0.18
Gauly et al., 2001			Elements of Docility test (illustrative traits shown) and categorical score	
	German Angus (249)	8 months (×2)	Range across elements	0.0–0.61 ± 0.17
			Time taken for separation from penmates (PH) (s)	Test 1: 0.03 ± 0.05
				Test 2: 0.02 ± 0.05
			Docility score test 1 (1–5: calm-very excited)	Pre-handling: 0.13± 0.11
				Handling: 0.61± 0.17
			Docility score test 2	Pre-handling: 0.11 ± 0.07
				Handling: 0.18 ± 0.07
	Simmental (206)	8 months (×2)	Range across elements	0.0–0.59 ± 0.41
			Time taken for separation from penmates (s)	Test 1: 0.16 ± 0.07
				Test 2: 0.38 ± 0.22
			Docility score test 1 (1–5: calm-very excited)	Pre-handling: 0.17± 0.12
				Handling: 0.55 ± 0.15
			Docility score test 2	Pre-handling: 0.35 ± 0.21
				Handling: 0.52 ± 0.20
Phocas et al., 2006	Limousin heifers (2781; 102 sires)	10–14 months	Docility test	0.18 ± 0.01
Fordyce et al., 1996	Bos inducus crosses (485)	Weaning	Flight distance	0.40 ± 0.15
		12 months		0.32 ± 0.14
	12 months: (312)	24 months		0.70 ± 0.23
Benhajali et al., 2009	Limousin (1,271; 65 sires)	8 months	Flight distance: Response to human approach (1–6: come near-charge)	0.17 ± 0.07–0.09

Sample size shown in parentheses in column with breed.

Some methodological differences may also explain the variation among estimates of heritability. In most cases, objective measures have higher heritabilities than more subjective scores (e.g., Burrow and Corbet, 2000; Benhajali et al., 2010). As expected, repeated measures result in higher heritabilities than a single measure (Burrow and Corbet, 2000). It is also apparent that heritability estimates decline with age at scoring. This may be due to habituation to the handling situation, which means that animals which show notable differences in temperament from group-mates when young gravitate toward the calmer end of the spectrum as they age, probably reducing both the genetic and phenotypic variation in the population. A reduction in phenotypic variation may also be expected through repeated testing of animals in a short period of time, as repeated handling reduces response intensity [as has been shown for flight speed (Burrow and Corbet, 2000; King et al., 2006)]. The influence of familiarity with humans on responsiveness is also shown by the effect of rearing intensity, whereby animals reared indoors are typically more docile than those reared under range conditions (Boivin et al., 1994). There may also be sex effects, with some finding that bulls are more excitable that cows (Burrow et al., 1988), but other studies have shown heifers to be more excitable than bulls (Voisinet et al., 1997a; Hoppe et al., 2010) or no difference (e.g., Cafe et al., 2010).

***Handling—dairy cattle***. There is also a range of heritabilities for milking temperament in dairy cattle from low to moderate with an unweighted mean of 0.19 (range 0.07–0.53) (Table 4). The larger number of records used in these studies ought to reduce measurement error, but compared to the heritabilities for beef cattle handling temperament measures, those for dairy cattle are typically lower. This may be due to the fact that individual farmers score their own dairy cows, and there may be lower inter-observer reliability than among trained assessors (the norm for beef cattle). Alternatively, there may be inherently low variation in dairy cattle temperament.

**Table 4:** **Heritability estimates for dairy cattle milking temperament**.

**References**	**Breed and sample size**	Measure^*^	**Heritability ± *SE***
Dickson et al., 1970	Holstein (1017)	Milking temperament (1–**4; quiet to restless**)	0.47
Wickham, 1979	Friesian (~6300)	Milking temperament (occasionally to often unsatisfactory)	0.11 – 0.12
	Jersey (~7800)		0.09 – 0.11
Sharma and Khanna, 1980	Dairy crossbreds (319)	Milking temperament (1–**4; quiet to restless**)	0.19
Lawstuen et al., 1988	Holstein (12,646)	Milking temperament (**1**–50: excitable-docile)	0.12 ± 0.02
Visscher and Goddard, 1995	Holstein Friesian (14,596)	Milking temperament (1–**5** good to poor)	0.22 ± 0.03
	Jersey (4695)		0.25 ± 0.06
Cue et al., 1996	Holstein (59,623)	Adaptability (how soon the animal settles into milking routine after calving: **1**–9: slowly to quickly)	0.111 ± 0.015
		Shed temperament: temperament of the animal during milking: **1**–9 vicious to placid)	0.137 ± 0.015
	Jersey (45,396)	Adaptability	0.179 ± 0.015
		Shed temperament	0.172 ± 0.015
	Ayrshire (6,599)	Adaptability	0.357 ± 0.06
		Shed temperament	0.333 ± 0.06
Schrooten et al., 2000	Holstein Friesian (656 bulls)	Milking temperament (1–9; direction not stated)	0.15
Pryce et al., 2000	Holstein Friesian (44,672)	Milking temperament (**1**–9: nervous-quiet)	0.07 ± 0.001
Hiendleder et al., 2003	Holstein (16 grandsires; mean sons: 54.5)	Milking temperament (1–9; direction not stated)	0.07
Sewalem et al., 2011	Holstein (1,940,092)	Milking temperament (**1**–5; nervous-calm)	0.13 ± 0.014

All animals were scored as adults.

* For milking temperament, figure in bold indicates score for most “restless/excitable/nervous” behavior.

***Other traits***. The studies of aggression and dominance with an adequate sample size appear to show that these traits have a low heritability (Table 5). However, for maternal traits, there is a range of heritability from low to moderate. This variation may reflect the quality of the trait definition, but does suggest that selective breeding could improve maternal temperament.

**Table 5:** **Estimates of heritability for traits other than handling**.

**DOMINANCE/AGGRESSION**				
Beilharz et al., 1966	Holstein (105) + Guernsey (8)	Adult	Dominance	0.40
Dickson et al., 1970	Holstein (1017)	Adult	Dominance	0.0
Phocas et al., 2006	Limousin (2781)	Youngstock	Maternal temperament	0.06 ± 0.02
Sartori and Mantovani, 2010	Valdostana (5981)	Adult	Fighting ability (winning): All fights	0.078
			Best result of each year	0.098
**MATERNAL TEMPERAMENT**				
Brown, 1974	Hereford (162)	Adult	Maternal temperament score	0.32
	Angus (266)			0.17
Morris et al., 1994	Bos taurus (2121; 486 sires)	Adult	Maternal temperament	0.09 ± 0.03
Phocas et al., 2006	Limousin (1502)	Youngstock	Maternal temperament	0.36 ± 0.06

A review of studies estimating heritability of temperament traits suggest that handling temperament traits have moderately high heritabilities that should allow them to be included in multi-trait selection programmes. Recent work on a larger scale and across different breeds has confirmed and extended earlier work by Burrow (1997). The estimates are similar to the heritability of some of the productivity traits which are primary targets for selection in the cattle sector [e.g., milk yield: 0.25 (Emanuelson et al., 1988); 0.27 (Woolliams, 1989)]. The variation in the heritability estimates is high in some cases, but may be due to variation between observers or the type of protocol used, which could be overcome with training of assessors and the creation of precise protocols.

### Relationship between temperament traits and other traits

In this section, the relationship between temperament traits, and productivity, health and fitness traits are reviewed. Some studies have investigated the mechanisms underlying these correlations. (See also Supplementary Material Tables A1–A7 for a list of papers and results).

#### Beef cattle

***Temperament, bodyweight, and growth***. Correlations between response to handling and weights at key ages (birth, weaning, yearling, and final weights) have been investigated. Generally, genetic and phenotypic correlations with temperament traits are low for weights from birth to one year of age, with high variation among estimates (e.g., Burrow, 2001; Prayaga and Henshall, 2005; Phocas et al., 2006). However, in a study with large numbers of animals, Sant'Anna et al. (2012) found unfavorable genetic and phenotypic relationships between weaning weight and flight speed in *Bos indicus* (Nellore) cattle, showing that animals with fast speeds had lower weights. Similarly, in a large study with *Bos taurus* cattle, Reinhardt et al. (2009) found that animals showing more excitable temperament scores in a chute test were phenotypically more likely to have a lower bodyweight on entry to a feedlot. Beyond the yearling stage, a number of studies with smaller numbers of cattle have shown phenotypic correlations between calm temperament and higher slaughter weights in both *Bos indicus* and *Bos taurus* breeds (chute score: Reinhardt et al., 2009; Cafe et al., 2011b; flight speed: Cafe et al., 2011b). However, a number of authors report contrasting relationships or different results in different animal populations within the same study (Burrow and Dillon, 1997; Burrow, 2001; Prayaga and Henshall, 2005). It is not clear why these studies had different results. They were based on a population of *Bos indicus* × *Bos taurus* cross-breds, in contrast to the other studies which used Bos taurus or Bos indicus breeds, but the differences may also be due to the specific test conditions. Some studies also report higher correlations with one measure over another (e.g., chute score higher than flight speed: Turner et al., 2011) but others find similar results for different measures (e.g., Hoppe et al., 2010; Cafe et al., 2011b). This suggests that interactions between breed and local contexts affect estimates.

The relationship of handling temperament with growth rate, rather than weight at a certain age, has also been investigated. Growth rate or daily gain are likely to be more accurate assessments, as they obviously take into account variation in initial bodyweight. Growth rates have also been shown to have unfavorable phenotypic relationships with temperament, indicating that cattle with excitable temperaments grow more slowly (*Bos indicus*: Voisinet et al., 1997a; Petherick et al., 2002; Cafe et al., 2011b; Sant'Anna et al., 2012; *Bos taurus*: Voisinet et al., 1997a; Fell et al., 1999; Müller and von Keyserlingk, 2006; Reinhardt et al., 2009; Turner et al., 2011). Estimations of genetic correlations often have large standard errors, but also show generally that more excitable animals tend to have slower growth (Hoppe et al., 2010; Sant'Anna et al., 2012). Phenotypic measures of feed efficiency also show a similar relationship, with lower efficiencies associated with high flight speed (Petherick et al., 2002; Cafe et al., 2011b). However, residual feed intake (RFI), studies have shown a low but negative genetic and phenotypic correlation of temperament with RFI values or no correlation with flight speed (Nkrumah et al., 2007; Elzo et al., 2009; Rolfe et al., 2011), with low (efficient) RFI scores associated with higher flight speeds. The correlations are low, indicating that the traits can be considered independent.

There is a similar picture for carcass weights. Excitable temperament, as measured objectively or subjectively by speed of movement from a chute, is genetically and phenotypically associated with lower carcass weights in both *Bos indicus* and *Bos taurus* animals but the relationship may not be present in all cohorts or breeds of animals (flight speed: Burrow and Dillon, 1997; Nkrumah et al., 2007; Cafe et al., 2011b; response to release from chute: Reinhardt et al., 2009). An unfavorable genetic correlation between temperament and carcass weight has also been reported, although the standard errors are large (Nkrumah et al., 2007).

Overall the data strongly suggests that animal growth and efficiency is unfavorably associated with behaviors in which the underlying trait is fearfulness of humans and/or of confinement. This may be because a fearful personality trait affects the animal in many situations that reduce its ability to eat sufficient feed, or that it responds more strongly to fear-inducing events than calmer animals, thereby reducing the energy available for growth (Petherick et al., 2002). Alternatively, the adverse response to handling may be long-lasting and reduce growth overall (MacKay et al., 2013). The genetic correlations are not strong, however, which suggests that selection for growth, final weight or efficiency will not have a dramatic impact on temperament. The general picture that poor temperament reduces productivity suggests that improvement of temperament will have a positive impact on animal welfare as well as farm profitability.

***Temperament and reproduction***. A number of studies have assessed the relationship between male and female reproductive characteristics and handling temperament traits. Scrotal circumference is often used as a measure of male and female reproductive performance. Low and negative genetic and phenotypic relationships with temperament have been reported suggesting that excitable animals have low scrotal circumference (response to corral/human presence: Barrozo et al., 2012; flight speed: Burrow, 2001; Sant'Anna et al., 2012). For females, Phocas et al. (2006) found significant genetic correlations showing that docile heifers had a lower age at puberty and higher fertility than less docile heifers, but other measures of fertility and reproductive function were not associated with temperament. A weak favorable genetic correlation between docility and maternal behavior was also found by Phocas et al. (2006), indicating that more docile animals had better maternal behavior, but this relationship was not confirmed by Turner et al. (2013) studying a wider range of maternal behavior traits. Other associations between temperament and reproductive traits are poorly studied but appear to be weak and variable in their direction. Burrow et al. (1988) found that calm cows were more likely to show behavioral signs of estrus in the presence of a human observer than excitable cows. Turner et al. (2013) found that cows which respond calmly to pre-calving handling produce slightly heavier calves that grow faster to weaning. It must be concluded however, that the weak relationships suggest either that the traits are largely independent, or that selection for reproductive traits is likely to have favorable but small effects on temperament.

***Temperament and stress physiology***. The physiological basis for the effect of temperament on productivity has been investigated in a number of studies. Differences in baseline levels of cortisol have been shown, with excitable animals having higher levels than calm animals (Fell et al., 1999; Curley et al., 2006b; King et al., 2006; Cafe et al., 2011a). Curley et al. (2008) looked at the response in detail and showed that despite having higher baseline levels, the excitable animals showed a blunted adrenal response to challenge compared to calm animals, indicating an elevated basal adrenal function that is often associated with chronic stress. Similarly, excitable animals have higher levels of epinephrine (a hormone associated with the sympathomedullary system) in baseline measures and following challenge such as transportation (Curley et al., 2006b; Burdick et al., 2011). These findings provide an explanation for the possible relationship between temperament and health discussed below.

***Carcass traits and meat quality***. In the *post-mortem* period in a normal animal, stored body energy in the form of glycogen is converted into lactate, which reduces muscle pH. Low lactate levels (and higher pH) are associated with tough meat (Maltin et al., 2003). As stress leads to a reduction in the levels of glycogen in muscle, it can reduce the levels available for conversion to lactate, thus affecting meat quality. This is particularly important in the pre-slaughter period when animals are transported and handled (King et al., 2006), events which excitable animals respond to adversely, as discussed above. Thus, the potential relationship between temperament and meat quality has important implications for animal welfare and farmer profit if payment based on meat eating quality becomes more widespread. A number of studies have shown a relationship between temperament and meat quality. The meat from excitable animals has higher shear force indicating lower tenderness than calmer animals as assessed by flight speed, chute test score and a combination of the two (Voisinet et al., 1997b; Reverter et al., 2003; Kadel et al., 2006; King et al., 2006; Cafe et al., 2011b; Hall et al., 2011). This relationship appears to be stronger at the genetic than the phenotypic level (Reverter et al., 2003; Kadel et al., 2006). A high carcass ultimate pH is also associated with poor temperament (Petherick et al., 2002; King et al., 2006) as is cooking loss (Kadel et al., 2006). However, there appears to be no phenotypic association between meat quality and temperament in frequently handled *Bos taurus* animals (Turner et al., 2011). The relationship between stress, pH and meat tenderness is not straight-forward, as the effects of acute and chronic stress on muscle physiology depend on a number of other factors such as post-mortem meat processing practices (King et al., 2006), which may explain some of the phenotypic variation.

***Temperament and health***. Chronic stress is known to have an immunosuppressive effect. However, there is only limited evidence that temperament is associated with clinical health parameters. For example, Fell et al. (1999) found that calm animals are less likely to be hospitalized in feedlots than excitable animals, and Reinhardt et al. (2009) showed that mortality rates were higher in excitable than calm steers. However, Burrow (2001), Prayaga (2003), and Prayaga and Henshall (2005) did not find significant relationships between temperament and counts of ticks or flies and fecal egg counts. Reinhardt et al. (2009) did not find any effect of temperament on number of respiratory treatments required or on incidence of lung lesions at slaughter. There is more evidence of a link between temperament and health at the level of immune function. A number of researchers have investigated a possible link between higher cortisol levels shown in animals with excitable temperaments and possible suppression of immune function. It has been reported that the innate immune system of calm animals shows more resistance to microbial invasion after a stressful challenge (transportation) (Hulbert et al., 2011). In contrast, calm beef steer calves had lower IgM levels than excitable calves (Fell et al., 1999; Burdick et al., 2009), but heifer calves showed the reverse pattern (Burdick et al., 2009). It is normally expected that higher immunoglobulin levels in young animals is beneficial in mounting a response to disease challenge.

#### Dairy cattle

Less work has been done on correlations between temperament and other traits in dairy cattle (See Supplementary Material Table B1). Research suggests that animals showing calm temperaments have better yields (Drugociu et al., 1977; Lawstuen et al., 1988; Breuer et al., 2000) and faster milking speed (Lawstuen et al., 1988; Sewalem et al., 2011). There is a positive relationship between temperament and survival in the herd, such that calmer cows are less likely to be culled (Haile-Mariam et al., 2004; Sewalem et al., 2010). There are also positive effects on health, with better resistance to mastitis, lower udder edema and better general health from animals with calmer temperaments (Lawstuen et al., 1988). However, there are conflicting reports on the relationship between temperament and somatic cell count (Fulwider et al., 2007; Sewalem et al., 2011). A strong genetic correlation between ease of calving and calm temperament was shown by Lawstuen et al. (1988) (0.48 ± 0.18), but in general, low phenotypic correlations have been reported for calving ease as well as other fertility traits, with high standard errors for the estimates reported (Lawstuen et al., 1988; Haile-Mariam et al., 2004; Sewalem et al., 2011).

### Consequences of selection for production on temperament

It would appear the inclusion of temperament in selection indices for both beef and dairy would have benefits for productivity and also animal welfare although many of the phenotypic associations between temperament and economic traits require further investigation at the genetic level. In beef cattle, calmer animals grow faster and have better feed conversion rates. Meat quality is better in calmer animals, and there may be benefits in terms of health and reproduction. In dairy cattle, milk production and milking speed is higher in calmer animals. Survival is higher in calmer animals, perhaps because farmers are more liable to cull animals that are difficult to milk. The health and fertility benefits are less clear in dairy animals.

In beef cattle, the low genetic correlations between productivity and temperament traits suggest that while selection for efficiency and growth would improve temperament, the correlated response to selection will be low. However, this also implies that current selection goals focussed on productivity alone will result in only a slow improvement in temperament. This may justify placing selection pressure on temperament itself in order to achieve more significant genetic progress in behavior and welfare which may be especially desirable for *Bos indicus* animals (Sant'Anna et al., 2012). Inclusion of temperament into a selection index would result in a reduction in selection pressure on other economically important productivity traits, and the implications of this would need to be quantified and considered.

### Molecular approaches: QTLs and GWAS

Over the last 30 years there has been a great deal of work worldwide to investigate the molecular genetic basis of a wide range of traits of interest in livestock production. This has included studies intended to detect quantitative trait loci (QTL), which are loci explaining a portion of the variation in traits of interest, as well as work to develop increasingly dense genome maps for farm livestock, and studies investigating associations between molecular genetic markers and traits of interest.

QTLs which influence behavioral traits have been found in a number of breeds (Table 6). Studies have shown significant or indicative QTL for a number of behavioral traits. Chromosomes 1, 8, 9, 16, and 29 are implicated across studies, although QTLs affecting behavior have been found on other chromosomes as well. Glenske et al. (2011) found an association between a candidate gene DRD4 on chromosome 29 and performance in the docility test. DRD4 is a dopamine receptor gene involved in curiosity and novelty seeking in mammals (Rubenstein et al., 1997). A database containing information on behavioral QTLs can be found at www.animalgenome.org/cgi-bin/QTLDB/index.

**Table 6:** **Studies identifying QTLs affecting behavior**.

**References**	**Breed**	**Test**	**Chromosome**	**Position**	**Flanking markers**
Spelman et al., 1999	Holstein Friesian and Jersey	Milking temperament (1–9: vicious-placid)	4		TGLA215
Schmutz et al., 2001	Beef cattle	“Temperament” (movement on a weigh scale in a race)	1	14	BMS574
			5	29	RM103
			9	44	ILSTS013
			11	57	ILSTS036
			14	19	RM180
				35	ILSTS008
			15	12	ADCY2
		“Habituation” (difference in response to two repeats of above test)	1	14	BMS574
			5	29	RM103
			9	44	ILSTS013
			11	57	ILSTS036
			15	12	ADCY2
Hiendleder et al., 2003	Holstein	Milking temperament (1–9)	5^*^	136	
			18^*^	105	
			29^*^	20	
			XY^*^	0	
Wegenhoft, 2005	Brahmanx Angus	Disposition (1–5: calm to crazy)	1^*^	37	DIK70-PIT17B7
	Mendelian model		4	46	TEXAN17-LAMB1
			8	0	BMS1864-BM3419
			9	72	BM6436-BM4208
			16	79	INRA013-BMS462
			18^*^	43	BL1016-BM8151
Boldt, 2008	Popn 1: Brahman/Nellore × Angus	Disposition (1–5: calm to crazy)	8	3 cM	BMS1864-CTSB
			8	2 cM	BMS1864-CTSB
	Parent of origin model				
	Popn 2: Angus × Nellore Mendelian model	Aggressiveness (toward humans when held in a raceway: 1–9 non-aggressive – extremely aggressive)	3	45 cM	BM7225-ILSTS64
			6^*^	1 cM	CSSM22-CSSM34
			12	20 cM	BMS2252-RM094
			29^*^	21 cM	BMC3224-BMS764
		Flightiness (1–9: quiet to flighty)	12^*^	22 cM	BMS2252-RM094
		Overall disposition (weaning)	12^*^	22 cM	BMS2252-RM094
		Overall disposition (yearling)	26^*^	33 cM	IDVGA59-HEL11
		Overall disposition (calving)	16^*^	70 cM	INRA48-BM3509
Esmailizadeh et al., 2008	Limousin × Jersey	Docility	2	5.6 cM	–
Gutierrez-Gil et al., 2008	Charolais × Holstein	Flight from feeder (distance moved when approached at feeder)	20^*^	64 cM	DIK15-BM5004
			25^*^	30	BM737-INRA222
			29	65	DIK94-MNB101
		Flight from feeder in repeated test	28^*^	0	BP23
			29^*^	66	DIK94-MNB101
		Sociality (locomotion in response to social separation)	16	0	BM121
		Habituation of above trait	6^*^	3	DIK5076-BM1329
			8^*^	115	DIK75-CSSM47
			9^*^	69	BM888-CSRM60
			19	40	BMS2142-CSSM65
			21^*^	65	HEL10-TGLA337
		Standing alert (response to social separation)	16	87	HUJ625-DIK4011
		Standing alert in repeated test	19^*^	72	CSSM65-ETH3
		Habituation of above trait in repeated test	1^*^	0	BM6438
			4	69	MAF50-DIK26
			11^*^	44	ILSTS100-IDVGA3
		Vocalization response to social separation	7^*^	41	RM6-BM1853
			16	49	ETH11-BM719
			18	21	IDVGA31-ABS13
		Vocalization in repeated test	9^*^	31	BM2504-UWCA9
			19^*^	72	CSSM65-ETH3
			25	33	BM737-INRA222
			26^*^	6	ABS12-HEL11
		Habituation of above trait in repeated test	1^*^	142	BNS4044
			4	68	MAF50-DIK26
			7^*^	93	ILSTS006-INRA53
			10^*^	43	BMS528-TGLA378
			29	31	RM44-MNB166
Glenske et al., 2010	German Simmental and German Angus	Weighing test (response to being weighed)	1	8	Allele 169 of BMS1928 (German Simmental)
					Allele 153 BMS574 (German Angus)
		Restraint (docility test)	1	15	Allele 153 BMS574 (German Simmental)
Glenske et al., 2011	German Angus	Temperament—response to entering a weigh scale	29	15.3	ILSTS081

* Significance at “suggestive” level (p < 0.05 chromosome-wide). Loci without superscripts are significant (P < 0.01 at chromosome-wise level or genome-wide).

However, while there are a few traits of interest in livestock that are largely determined by genotype at a single locus or a few loci, there are many more traits of interest that appear to be polygenic in nature, and influenced by many, often hundreds, of loci (Hayes et al., 2009). Moreover, there are often rather few genes that have a large effect on these polygenic traits, and many more that individually have a small effect.

Increasingly dense genome maps are available for livestock with tens or hundreds of thousands of single nucleotide polymorphisms (SNPs) measured throughout the genome. These, coupled with automated platforms for genotyping on so-called SNP “chips,” allow genome-wide association studies (GWAS) to be done relating markers to traits of interest, including temperament traits. In beef cattle, a study of temperament and meat quality in Nellore-Angus beef cattle found an association between response to social separation in a pen and a gene regulating sodium ion transport, indicating a difference in nervous system responsiveness (Hulsman Hanna et al., 2014). Additionally, a study in Brown Swiss cattle identified regions with high influence on temperament and aggression on chromosomes 4, 8, and 14 (Kramer et al., 2014). As mathematical techniques are developed that will allow evaluations across breeds and as costs of genotyping fall, more studies that include the assessment of temperament traits are likely.

The availability of dense genome maps and rapid, increasingly affordable genotyping has altered the paradigm for application of molecular genetics in livestock breeding, for many traits of interest. Rather than relying on genotypes at a few loci to predict genetic merit, predictions are increasingly based on information from tens or hundreds of thousands of SNPs throughout the genome. The prediction of genetic merit itself relies on GWAS in a “reference population” of animals—large populations of relevant animals that have both molecular genetic and phenotypic information available. GWAS followed by genomic selection is thought to be a particularly useful approach to improving traits that are difficult, expensive or time-consuming to measure, such as temperament traits. Once the trait has been measured in the reference population, candidates for selection from other similar populations need only be genotyped to predict their genetic merit for temperament (though associations need to be re-estimated periodically). Direct genomic breeding values (dGEBVs) can be predicted from molecular genetic information alone, but increasingly these are combined with EBVs derived from phenotypic records on candidates for selection and their relatives, to enhance accuracy. Figure 1 illustrates the steps involved in prediction of genomic and conventional breeding values.

Both the dairy and beef industries are already using, or moving toward the use of, genomic estimated breeding values (GEBVs). Until recently, much of the genomic research has focussed on productivity traits, meat quality and reproductive traits (see Hayes et al., 2009; and Garrick, 2011 for reviews). This may be because the number of animals with phenotypes required is very large. The need to do the analysis on each breed individually, and the costs of phenotyping and genotyping relative to the perceived benefit of assessing temperament traits are likely to be (at least short term) constraints on the use of this technique. However, many phenotypes can be assessed in each study, allowing temperament to be assessed alongside traits seen to be more economically important.

### The use of temperament traits in selection programmes

From the research reviewed above, it would appear that many of the building blocks for selection indexes that include temperament traits exist: the traits can be defined and measured, heritability estimates are available from studies on large numbers of animals in which the traits are carefully measured, and these are similar to heritabilities of many other traits currently under selection. Genetic correlations for a number of temperament traits with productivity measures have been estimated. There is not always consensus across the studies, but some of the larger studies provide strong evidence of favorable genetic correlations.

However, temperament is not often included in breeding indexes. In dairy cattle breeding, EBVs for milking temperament are available as stand-alone EBVs, or information on bulls is available in sub-index scores for “workability” that includes milking speed for some countries. The situation for beef cattle is similar. Although the correlation between handling temperament and growth and meat quality suggest that including temperament in a selection index would be beneficial from a profit and welfare point of view, it is not currently used. Animals may be excluded based on their raw score. Stand-alone EBVs are available (such as flight time for some *Bos indicus* breeds in Australia and North America, and docility scores for British and European breeds in some countries), but the trait is not currently included in a selection index (Johnston, personal communication).

## Constraints and barriers

There are a number of possible technical and producer motivational reasons why temperament traits are not incorporated into selection indexes. A major technical barrier to the use of temperament traits in selection indexes is the need for economic values to allow the trait to be weighted in a selection index. However, it should be possible to derive an economic weight for temperament from the effects that it has on meat quality and growth in beef cattle and the additional labor costs incurred from an animal that is difficult to handle or a cow that is slow or difficult to milk. This has indeed been done for *Bos taurus* cattle by a team in the US (Busby et al., 2006), but other estimates of economic values are lacking. Another issue is the lack of complete information on genetic and phenotypic correlations of temperament with all the parameters that could be used in selection indexes. For some breeds in some countries, correlations between productivity, meat quality, some fertility traits and temperament have been investigated, but by no means all. New traits are also being incorporated into selection indexes, such as calving ease, and the correlation of this trait with temperament must be determine before both traits can be included. Herd or industry scale and level of organization at the national level may also be important. The pattern of uptake suggests that in regions or countries where a breed is numerous and the breed society or governmental body is well-organized enough to provide support for the recording and evaluation of temperament traits, handling temperament traits may be evaluated. It may be that addition of temperament to an existing selection index has little impact on the overall response, but it should be examined.

Producer motivational factors are also involved. It is clear that temperament is generally poorer in *Bos indicus* breeds than *Bos taurus* animals, which may explain the greater motivation to assess temperament in *Bos indicus* animals. In some *Bos taurus* breeds, the perception of the breed as being flighty or difficult to handle appears to motivate the breed society to make genetic evaluations on temperament measures such as docility, to improve the trait and improve the popular image of the breed. Additionally, as the response to handling can be modified by repeated handling and habituation to the proximity of humans, the farming system used in any country or region will influence the necessity or motivation of producers to use genetic selection mechanisms to deal with temperament issues. On smaller farms, which are typical of much of Europe, animals arguably experience a higher level of human contact during pasture rotations or seasonal housing than the larger extensive rangeland or feedlot systems more typical of Australia and America. Extreme responses to handling may decline as repeated exposure allows the animal to habituate to human proximity and the handling process. Thus, husbandry conditions may reduce the necessity to use genetic selection to improve temperament. There is also the perception amongst some European producers that some degree of reactivity in animals is desirable, as it promotes survival and competitiveness. Clearly, farm extension and advisory work is needed to inform producers of the negative effects of poor temperament on productivity and profitability.

## The future: moving forward and overcoming constraints

As it appears important for welfare and economic reasons to improve handling temperament, we need to facilitate the use of these traits in selection indexes. Research has shown that it is possible to clearly define and accurately record these traits. An increased understanding of the biological basis of these traits will also improve progress. Across the globe, several breed societies and countries have developed EBVs based on farmer-recorded assessments of temperament in dairy cattle and different types of temperament tests in beef cattle. In beef cattle, the lack of a single measure of temperament in beef cattle may impede progress. In the dairy sector in particular, standardization of recording—initially for milk-related traits, and latterly for a much wider set of traits—has helped to underpin improvement in these traits (along with the widespread use of artificial insemination, well-designed breeding programmes usually based on progeny testing, and development of statistical techniques to improve the prediction of genetic merit of animals within and across countries).

Genomic selection may provide an important opportunity for increased use of temperament traits, as increasingly, the prediction of the genetic merit of farm animals will include molecular genetic information. As the cost of genotyping falls, and the predictive power of the information increases, the rate-limiting step to application is likely to be the lack of high-quality records of traits of interest, or phenotypes, both to investigate associations between genotypes and traits of importance in the first place, or to allow ranking of candidates for selection. Temperament traits would therefore need to be measured in comprehensively recorded reference populations, and the correlations between these traits and all others estimated. Within the foreseeable future, GEBVs could also be based on complete DNA sequence information, at least for potentially influential animals.

We should also look to the inclusion of temperament traits other than the response to handling. The correlations between tests measuring response to handling and responses in other contexts can be low. Therefore, selection on the basis of chute test or flight speed may have little impact on traits which are contextually different, such as intra-specific aggressiveness, sociability and maternal defensiveness (e.g., Turner et al., 2013). This review suggests little work has been done on personality traits other than handling temperament, and yet selection for maternal ability and appropriate levels of aggression and sociability and flexibility may be important in terms of animal health and welfare, and also in terms of farm efficiency. Achieving improvements in the latter traits will require the development of automated methods for their measurement. This may come from technologies such as the use of automatic measurement of eye-white (Core et al., 2009), thermal imaging of body areas that show alteration due to stressful events or from other methods, or the use of data collected from activity monitors used to detect oestrus that can be used to characterize personality traits (MacKay et al., 2013). Automatic methods for assessment of meat quality in abattoirs will provide further incentives to improve temperament in beef cattle.

However, even when more efficient methods of phenotyping behavior and other correlated traits are developed, implementation of selection will continue to require that the industry recognizes the need for temperament traits to be used in breeding programs. The case for inclusion seems clearest for handling temperament in beef and dairy, but other traits require more research. Understanding the value of selection for temperament traits will be facilitated by continued effort to clarify and quantify the full range of economic and welfare implications of poor temperament. The primary focus of selection pressure primarily on “output” traits such as carcass weight or milk yield will most likely change in future, as new traits such as RFI are likely to be included in selection goals, particularly in beef, but possibly also in dairy cattle, responding to the need to reduce greenhouse gas emissions from agriculture or the economic necessity for improved feed use efficiency. Animal behavior, particularly behavioral responses to stress, has been hypothesized to be a determinant of feed use efficiency (Richardson and Herd, 2004). In reality, there is a paucity of information on how temperament correlates with feed use efficiency and the information available is contradictory (Petherick et al., 2002; Nkrumah et al., 2007; Elzo et al., 2009; Cafe et al., 2011b; Rolfe et al., 2011). Therefore, it has yet to be established whether improving feed use efficiency will bring with it improvements in temperament, but the low correlations shown in this review suggest that progress will be slow. Changes in some biological systems in response to improvements in feed use efficiency, such as a down-regulation of the rate of endogenous protein turnover, could compromise the animals' ability to respond to stress (Baldwin et al., 1980) leading to changes in behavior and implications for welfare. Given the global interest in improving feed use efficiency in cattle, there is therefore a need to understand the role of temperament as a driver of efficiency and, conversely, how changing feed use efficiency may impact on welfare through other routes.

As handling temperament and other behavioral traits clearly have economic value and animals that respond poorly to handling, and in other situations, suffer negative emotional and physical experiences, resulting in reduced welfare, it is clearly important to improve temperament. Genetic improvement will be important as well as investment in appropriate housing and handling systems. Genetic improvement may become more important against a background of increased herd size, intensification of beef and dairy enterprises and reduced availability of labor. Increased automation and advances in genomic techniques that allow identification of genetically superior animals once the markers have been located in training populations will contribute and quantitative methods will also continue to be important.

## Funding

Neither SRUC nor any of the authors received any financial support from a third party in the preparation of this manuscript. SRUC receives funding from the Scottish Government.

### Conflict of interest statement

The authors declare that the research was conducted in the absence of any commercial or financial relationships that could be construed as a potential conflict of interest.
