# Supplementary material for: Genetic selection for temperament traits in dairy and beef cattle
Source: Front Genet. 2014 Oct 21;5:368. doi: 10.3389/fgene.2014.00368 (PMC4204639; doi:10.3389/fgene.2014.00368)
Supplement: Supplementary file 1 [file Table1.DOCX]

Supplementary material

Table A1. Table showing genetic and phenotypic correlations between temperament traits and productivity for beef cattle (grouped by productivity trait). All weights are in kg.

| **PRODUCTIVITY** | | | | | | | | |
| --- | --- | --- | --- | --- | --- | --- | --- | --- |
| **Liveweight** | | | | | | | | |
| **Trait** | **Temperament measure†** | **Age and frequency of temperament measure** | **Breed (sample size in parentheses)** | **Age at which trait was measured** | **Correlation*** | | | **Author(s)** |
| Birth weight | Flight time | 1-9x ( 8-18 mo) | *Bos taurus* derived and  *Bos indicus* derived (~2555) | Birth | Genetic: -0.08 ± 0.01-0.18  Phenotypic: -0.03 ± 0.01-0.03 **NS** | | | Prayaga and Henshall, 2005 |
|  | Flight speed | 3-6x: 6-18 mo | Bos indicus derived (Belmont Red)  (1871) | Birth | Genetic: -0.03  Phenotypic: -0.03 | | | Burrow, 2001 |
| Weaning weight  Weaning weight (cont.) | Temperament : summed scores for fear responses to handling/confinement in a race | At weaning  12 mo  24 mo | Bos indicus cross (n=485) | Weaning | Genetic: 0.89 ± 0.46 **NS** | | | Fordcye et al 1996 |
|  |  |  | (485) |  | Genetic: -0.06 ± 0.48**NS** | | |  |
|  |  |  | (485) |  | Genetic: 0.36 ± 0.59 **NS** | | |  |
|  | Flight distance (minimum tolerated approach distance in a yard) | At weaning | (485) |  | Genetic: 0.33 ± 0.31 **NS** | | |  |
|  |  | 12 mo | (485) |  | Genetic: -0.03 ± 0.34 **NS** | | |  |
|  | Flight speed | 3-6x: 6-18 mo | Bos indicus derived (Belmont Red)  (n=1871) | Weaning: 6 mo | Genetic: 0.00  Phenotypic: -0.02 | | | Burrow, 2001 |
|  | Flight time | 1-9x 8-18 mo | Bos taurus derived and  Bos indicus derived (n~2555) | Weaning (193 d) | Genetic:0.01 ± 0.01-0.18 **NS**  Phenotypic -0.01 ± 0.01-0.03 **NS** | | | Prayaga and Henshall, 2005 |
|  | Flight speed | Yearling (495 d) | Nellore  (n=7402) | Weaning | Genetic: -0.12 ± 0.07  Phenotypic: -0.07 ± 0.01 | | | Sant’Anna et al., 2012 |
| Yearling weight | Temperament : summed scores for fear responses to handling/confinement in a race – weaning (calm-excitable) | Weaning | Bos indicus cross (n=485) | 12 mo | Genetic: 1.10 ± 0.53 **NS** | | | Fordcye et al 1996 |
|  |  | 12 mo | (n=485) |  | Genetic: -0.03 ± 0.53 **NS** | | |  |
|  |  | 24 mo | (n=485) |  | Genetic: 0.34 ± 0.63 **NS** | | |  |
|  | Flight distance (minimum tolerated approach distance in a yard (m)) | Weaning | Bos indicus cross (n=485) |  | Genetic: 0.31 ± 0.34 **NS** | | |  |
|  |  | 12 mo | (n=485) |  | Genetic: -0.02 ± 0.37 **NS** | | |  |
|  |  | 24 mo | (n=312) |  | Genetic: 1.12 ± 1.51 **NS** | | |  |
|  | Flight speed | 3-6x: 6-18 mo | Bos indicus derived (Belmont Red)  (n=1871) | 12 mo | Genetic: 0.01  Phenotypic correlation:  -0.05 | | | Burrow, 2001 |
|  | Flight time | 1-9x 8-18 mo | Bos taurus derived and  Bos indicus derived (n~2555) | 12 months | Genetic:0.0 ± 0.01-0.18 **NS**  Phenotypic 0.01 ± 0.01-0.03 **NS** | | | Prayaga and Henshall, 2005 |
|  | Docility score (**6.5**-17: aggressive to docile) | 10-14 mo | Limousin (n=2781) | 12 mo | 0.08 ± 0.09 **NS** | | | Phocas et al., 2006 |
| Weight on entry to feedlot | Disposition score (response on exit from chute1-**6**: calm to excitable) | 3-5x during feedlot finishing | Bos taurus (Continental and British)( n= 21,528) | On entry to feedlot | More excitable = lower initial BW; P<0.001 | | | Reinhardt et al., 2009 |
| Weight at 18 mo | Temperament : summed scores for fear responses to handling/confinement in a race – weaning (calm-excitable) | Weaning | Bos indicus cross (n=485) | 18 mo | Genetic: 1.06 ± 0.56 **NS** | | | Fordcye et al 1996 |
|  |  | 12 mo | (n=485) |  | Genetic: -0.11 ± 0.56 **NS** | | |  |
|  |  | 24 mo | (n=485) |  | Genetic: -0.02 ± 0.66 **NS** | | |  |
|  | Flight distance (minimum tolerated approach distance in a yard (m)) | Weaning | Bos indicus cross (n=485) |  | Genetic: 0.47 ± 0.36 **NS** | | |  |
|  |  | 12 mo | (n=485) |  | Genetic: 0.08 ± 0.39 **NS** | | |  |
|  |  | 24 mo | (n=312) |  | Genetic: 0.44 ± 0.43 **NS** | | |  |
| Weight at 24 mo | Temperament : summed scores for fear responses to handling/confinement in a race – weaning (calm-excitable) | Temp At weaning | Bos indicus cross (n=485) | 24 mo | Genetic: 0.94 ± 0.45 **NS** | | |  |
|  |  | 12 mo | (n=485) |  | Genetic: -0.04 ± 0.46 **NS** | | |  |
|  |  | 24 mo | (n=485) |  | Genetic: -0.20 ± 0.54 **NS** | | |  |
|  | Flight distance (minimum tolerated approach distance in a yard (m)) | At weaning | Bos indicus cross (n=485) |  | Genetic: 0.42 ± 0.29 **NS** | | |  |
|  |  | 12 mo | (n=485) |  | Genetic: -0.03 ± 0.33 **NS** | | |  |
|  |  | 24 mo | (n=312) |  | Genetic: 0.08 ± 0.34 **NS** | | |  |
| Cow weight | Flight speed | 3-6x: 6-18 mo | Bos indicus derived (Belmont Red)  n=913 | 32 mo and over | Genetic:-0.02  Phenotypic: -0.06 | | | Burrow, 2001 |
| Calving weight | Docility score (6.5-17: aggressive to docile) | 10-14 mo | Limousin heifers (n=2781; 102 sires) | ~24 mo | Genetic: -0.04 ± 0.11**NS** | | | Phocas et al., 2006 |
| **Liveweight at slaughter** | | | | | | | | |
| Final liveweight  Final liveweight (cont.) | Vigour of movement in a race (calm-excitable: 1-7) | Prior to slaughter | Bos indicus cross  (n=232) | 12-42 mo | | Phenotypic: -0.35; P<0.001 | Fordyce et al., 1985 | |
|  | Count of audible respiration when confined in a race |  |  | 12-42 mo | | Phenotypic: -0.22; P<0.01 |  |  |
|  | Temperament: sum of scores for fear responses to handling/confinement in a race (calm-excitable) |  |  | 12-42 mo | | Phenotypic -0.34; P<0.001 |  |  |
|  | Flight speed | 8-11x: ~12 mo | Bos indicus crossbreds  (n=96) | Approx 18 m | | Regression coeff: 40.45; P<0.05 | Burrow and Dillon, 1997 | |
|  |  |  | Bos indicus crossbreds  (n=119) | Approx 16 mo | | Regression coeff=  8.33; **NS** |  |  |
|  | Flight speed | 3-6x: 6-18 mo | Bos indicus crossbreds (Belmont Red)  (n=1871) | 18 mo | | Genetic: 0.00  Phenotypic: -0.03 | Burrow, 2001 | |
|  | Flight time | 1-9x 8-18 mo | Bos taurus derived and  Bos indicus derived (n~2555) | 524 d | | Genetic:0.0 ± 0.01-0.18 **NS**  Phenotypic -0.01 ± 0.01-0.03 **NS** | Prayaga and Henshall, 2005 | |
|  | Flight speed (m/s) | 2x? (10- 12 mo) | Bos taurus (Angus, Charolais, Hybrid) (n=302) | In 70 d trial (10-12 mo) | | Genetic: -0.57 ± 0.29  Phenotypic: -0.08 **NS** | Nkrumah et al., 2007 | |
|  | Disposition score (response on exit from chute1-**6**: calm to excitable) | 3-5x during feedlot finishing | Bos taurus (Continental and British) (n= 21528) | Final weight | | More excitable = lower final BW; P<0.001 | Reinhardt et al., 2009 | |
|  | Flight speed | During back-grounding at pasture  (5xin NSW)  (2x in WA) | Brahman (NSW popn) (n=164) | 17-19 mo | | Slope: -21.0 ± 5.0; P<0.001 | Cafe et al., 2011b | |
|  |  |  | Brahman (WA popn) (n=173) | 21-24 mo | | Slope: -20.9± 7.8; P<0.01 |  |  |
|  |  | During feedlot phase (9x in NSW)  (2x in WA) | Brahman (NSW popn) (n=164) | 17-19 mo | | Slope: -11.1±5.1; P<0.05 |  |  |
|  |  |  | Brahman (WA popn); (n=173) | 21-24 mo | | Slope: -18.3 ±7.5; P<0.05 |  |  |
|  |  |  | Angus (WA popn); ( n=20) | 22-26 mo | | Slope: -27.6 ± 13.1; P=0.06 |  |  |
|  | Crush score (1-**5**: calm to very nervous) | During back-grounding at pasture (6xin NSW) | Brahman (NSW popn); (n=164)  Brahman (NSW popn); (n=164)  (cont.) | 17-19 mo  17-19 m (cont.) | | Slope: -11.9 ± 6.9; P=0.09 |  |  |
|  |  | During feedlot phase (11x in NSW) |  |  |  | Slope: -30.0± 7.8; P<0.001 |  |  |

†Flight time is typically measured in s or s*100; flight distance in m. Scales for chute or other restraint tests and the docility test are shown with a character in bold indicating the excitable/nervous end of the scale.

*NS: author notes correlation as non-significant

Table A2. Table showing relationships between temperament traits and measures of growth and efficiency in beef cattle (grouped by trait). (All weights are in kg).

| **Growth and efficiency** | | | | | | |
| --- | --- | --- | --- | --- | --- | --- |
| **Trait** | **Temperament measure†** | **Age and frequency of temperament measure** | **Breed (sample size in parentheses)** | **Age at which trait was measured** | **Correlation*** | **Author(s)** |
| Liveweight gain (kg/d) | Flight speed | 8-11x: ~12 mo | Bos indicus crossbreds  (n=96) | Weaning to slaughter | Regression coefficient:  0.29; P<0.05 | Burrow and Dillon, 1997 |
|  |  |  | Bos indicus crossbreds  (n=119) | Weaning to slaughter | Regression coefficient: 0.08; **NS** |  |
| Growth rate (g/day) weaning to 18 mo  Growth rate (g/day) weaning to 18 mo (cont.) | Temperament : summed scores for fear responses to handling/confinement in a race – weaning (calm-excitable) | At weaning | Bos indicus cross (n=485) | Weaning-18 mo  Weaning-18 mo (cont.) | Genetic: 0.10 ± 0.45 **NS** | Fordyce et al., 1996  Fordyce et al., 1996 (cont.) |
|  |  | 12 mo | (n=485) |  | Genetic: -0.06 ± 0.48 **NS** |  |
|  |  | 24 mo | (n=485) |  | Genetic: -0.57 ± 0.60 **NS** |  |
|  | Flight distance (minimum tolerated approach distance in a yard (m)) | At weaning | Bos indicus cross (n=485) |  | Genetic: 0.14 ± 0.32 **NS** |  |
|  |  | 12 mo | (n=312) |  | Genetic: 0.20 ± 0.33 **NS** |  |
| Growth rate (g/day) weaning to 24 mo | Temperament : summed scores for fear responses to handling/confinement in a race – weaning | At weaning | Bos indicus cross (n=485) | Weaning-24 mo | Genetic: 0.27 ± 0.41 **NS** |  |
|  |  | 12 mo | (n=485) |  | Genetic: -0.02 ± 0.43 **NS** |  |
|  |  | 24 mo | (n=485) |  | Genetic: -0.56 ± 0.55 **NS** |  |
|  | Flight distance (minimum tolerated approach distance in a yard (m) | At weaning | Bos indicus cross (n=485) |  | Genetic: 0.18 ± 0.28 **NS** |  |
|  |  | 12 mo | (n=312) |  | Genetic: -0.01 ± 0.30 **NS** |  |
|  |  | 24 mo | (n=485) |  | Genetic: -0.19 ± 0.36 **NS** |  |
| Average daily gain (kg/d) | Chute test (1-**5**: calm-violently struggling) | 7-11 mo (monthly) | Bos taurus  (n=124) | 7-11mo to slaughter | Calm animals had 0.19 kg/d higher gain than excitable; P<0.05 | Voisinet et al., 1997b |
|  |  | 7-11mo (monthly) | Bos indicus cross (n=304) | 7-11mo to slaughter | P<0.05 calm animals had higher ADG |  |
| Average daily gain (kg.head/day) | ‘Temperament’: (calm and nervous: combination of flight time, crush score and willingness to move past humans to food) | 2x: weaning and 21d post-weaning | Angus x Hereford and Hereford (n=24, selected from a group of 209) | Finishing period 13-15 mo to slaughter  After 37 d  After 78d | R_s_=0.707; P<0.01  Calm animals (high flight times) had higher ADG than nervous animals:    Calm animals higher ADG (P<0.05)  Calm animals higher ADG (P<0.05) | Fell et al., 1999 |
| Daily liveweight gain (kg/d) | Docility: separated into pre-handling (PH) and handling (H) (low scores indicate calm temperament) | 8 mo (2 and 3 weeks post-wean) | Simmental (n=206)  German Angus (n=249) | ? | Test 1 PH: 0.07 ± 0.05  Test 1 H: -0.22 ± 0.21  Test 2 PH: -0.12 ± 0.07  Test 2 H: -0.12 ± 0.20 | Gauly et al., 2001 |
| Daily liveweight gain (pre-weaning) | Flight speed | Measures 3-6x; 6-18 mo | Bos indicus derived (Belmont Red)  (n=1871) | Birth to weaning | Genetic: 0.00  Phenotypic: -0.02 | Burrow, 2001  (paper also contains data on growth rate in wet and dry seasons |
| ADG birth to weaning | Flight time | 1-9x 8-18 mo | Bos taurus derived and  Bos indicus derived (n~2555) | ADG birth to weaning | Genetic:0.0 ± 0.01-0.18 **NS**  Phenotypic -0.02 ± 0.01-0.03 **NS** | Prayaga and Henshall, 2005 |
| Daily liveweight gain (post-weaning) | Flight speed | Measures 3-6x; 6-18 mo | Bos indicus derived (Belmont Red)  (n=1871) | Weaning to final | Genetic: 0.01  Phenotypic: -0.02 | Burrow, 2001 |
| ADG weaning to final | Flight time | 1-9x 8-18 mo | Bos taurus derived and  Bos indicus derived (n~2555) | ADG weaning to final (524 d) | Genetic:-0.12 ± 0.01-0.18 **NS**  Phenotypic 0.0 ± 0.01-0.03 **NS** | Prayaga and Henshall, 2005 |
| Average daily gain | Flight speed (m/s) | Day 0 in feedlot (~29 mo) | Bos indicus crosses (n=120) | Finishing: 29 mo at start  Day 0-45 in feedlot | r= -0.20 (P<0.05) | Petherick et al., 2002 |
|  |  |  |  | Days 0-70 | r= -0.19 (P<0.05) |  |
|  |  |  |  | Days 0-101 | r= -0.18 (P<0.05) |  |
|  |  | Day 21 in feedlot |  | Days 0 - 21 | r= -0.36 (P<0.01) |  |
|  |  | Day 45 in feedlot |  | Days 0-45 | r= -0.32 (P<0.01) |  |
|  |  | Day 70 in feedlot |  | Days 0-70 | r= -0.20 (P<0.05) |  |
|  |  | Day 101 in feedlot |  | Days 0-101 | r= -0.25 (P<0.01) |  |
| Average daily gain (kg/d) | Flight speed (m/s) | 4x (8-11 mo) | Angus crossbreds (n=61) | 8 mo | Quadratic relationship (highest FS lowest ADG)  R^2^= 0.14; (P<0.05) | Müller and von Keyser  lingk (2006) |
|  | Flight speed (m/s) | 2x? (10- 12 mo) | Bos taurus (Angus, Charolais, Hybrid) (n=302) | In 70 d trial (10-12 mo) | Genetic: -0.25 ± 0.25  Phenotypic: -0.26 (P<0.01) | Nkrumah et al., 2007 |
|  | Flight speed | Yearling | Nellore  (n=7402) | Weaning to yearling | Genetic: -0.13 ± 0.08  Phenotypic: -0.06 ± 0.01 | Sant’Anna et al., 2012 |
|  | Disposition score (response on exit from chute1-6: calm to excitable) | 3-5x during feedlot finishing | Bos taurus (Continental and British) (n=21,528) | Between feedlot entry and final wt | More excitable = lower ADG; P<0.001 | Reinhardt et al., 2009 |
| Average daily gain | Response to social separation (durations of types of behavior) | 1x (8-11 mo) | Angus crossbreds (n=61) | 8 mo | No. areas in test pen entered: high ADG more areas entered (r= 0.41; P<0.05), and showed more walking: (r= 0.50; P<0.01) and less time vigilant (r= -0.40; P<0.05) | Müller and von Keyser  lingk (2006) |
| Average daily gain | Chute score (1-**5**: calm to violently struggling) | 1x: 278 d | German Angus (n=706) | Calves (5-11 mo)  Calves (5-11 mo) | Genetic correlation  -0.13 ± 0.22 | Hoppe et al., 2010 |
|  |  | 1x: 263 d | Charolais (n=556) |  | -0.16 ± 0.12 |  |
|  |  | 1 x: 194 d | Hereford (n=697) |  | -0.58 ± 0.11 |  |
|  |  | 1 x: 233 d | Limousin (n=424) |  | -0.27 ± 0.27 |  |
|  |  | 1 x:202 d | German Simmental (n=667) |  | -0.34 ± 0.18 |  |
|  | Flight score (1-**4**: walk to jumping out) | 1x: 278 d | German Angus (n=706) |  | -0.04 ± 0.12 |  |
|  |  | 1x: 263 d | Charolais (n=556) |  | -0.29 ± 0.17 |  |
|  |  | 1 x: 194 d | Hereford (n=697) |  | -0.37 ± 0.11 |  |
|  |  | 1 x: 233 d | Limousin (n=424) |  | -0.41 ± 0.27 |  |
|  |  | 1 x:202 d | German Simmental (n=667) |  | -0.27 ± 0.14 |  |
| Average daily gain | Chute score (1-**6**: calm to dangerous) | 4x during fattening (15-18 mo) | Angus or Limousin crossbreds (n=144) | During fattening  (15-18 mo) | Calm cattle greater ADG;  P=0.05 | Turner et al., 2011 |
|  | Isolation score (1-**6**: response to isolation in a pen with handler: part of the docility test | 1 or 2x during fattening (15-18 mo) |  | Final finishing to carcass weight | Fearful cattle had a reduced ADG; P=0.02 |  |
| Average daily gain (kg) during growing/backgrounding at pasture | Flight speed | During back-grounding at pasture (5xin NSW)  (2x in WA) | Brahman (NSW popn); (n=164) | Growing phase: 6-15 mo | Slope:-0.02±0.01; P<0.05 | Cafe et al., 2011b  Cafe et al., 2011b  (cont.) |
|  |  |  | Brahman (WA popn); (n=173) | Growing phase: 6-21 mo | Slope: -0.05±0.02; P<0.05 |  |
|  |  | During feedlot phase  (2x) | Angus (WA popn); (n=20) | Growing phase: 8-24 mo | Slope: -0.16 ±0.04; P<0.01 |  |
|  | Crush score (1-**5**: calm to very nervous) | During backgrounding at pasture (6xin NSW) | Brahman (NSW popn); (n=164) | Growing phase: 6-15 mo | Slope: -0.04±0.02; P<0.05 |  |
| Average daily gain (kg) during feedlot finishing | Flight speed  Flight speed  (cont.) | During back-grounding at pasture (5xin NSW)  (2x in WA) | Brahman (NSW popn) (n=164) | Finishing phase: 15-19 mo | Slope: -0.08 ± 0.03; P<0.01 |  |
|  |  |  | Brahman (WA popn (n=173) | Finishing phase: 21-24 mo | Slope: -0.10 ± 0.06; P=0.07 |  |
|  |  | During feedlot finishing phase  (9x in NSW)  (2x in WA) | Brahman (NSW popn) (n=164) | Finishing phase: 15-19 mo | Slope: -0.07 ± 0.03; P<0.01 |  |
|  |  |  | Brahman (WA popn) (n=173) | Finishing phase: 21-24 mo | Slope: -0.08 ± 0.05; P=0.07 |  |
|  | Crush score (1-**5**: calm to very nervous) | During feedlot finishing (11x in NSW) | Brahman (NSW popn) (n=164) | Finishing phase: 15-19 mo | Slope: -0.12 ± 0.04; P<0.01 |  |
| ADG | Flight speed | 21-24 days and weaning | Brahman (n=116) | Young calves: d0-d24 of life | Correlation: -0.06; NS | Burdick et al., 2009 |
|  | Flight speed | Finishing phase (periodically) | Bos taurus (n=1141) | Finishing phase (8 mo to slaughter | Genetic:0.07 ± 0.27  Phenotypic: -0.04 ± 0.04 | Rolfe et al., 2011 |
| iFCE (inverse Feed conversion efficiency: mean daily gain/mean daily intake) | Flight speed | Day 0 in feedlot (~29 mo) | Bos indicus crossbreds (n=120) | Finishing: 29 mo at start  Day 0-45 in feedlot | r = -0.68 (P<0.05) | Petherick et al., 2002 |
|  |  |  |  | Days 0-101 in feedlot | r = -0.60 (P<0.05) |  |
|  |  | Day 21 in feedlot |  | Days 0-21 | r = -0.60 (P<0.05) |  |
|  |  | Day 45 in feedlot |  | Days 0-45 | r = -0.73 (P<0.01) |  |
|  |  | Day 70 in feedlot |  | Days 0-70 | r = -0.63 (P<0.05) |  |
|  |  | Day 101 in feedlot |  | Days 0-101 | r = -0.73 (P<0.05) |  |
| FCR(kg of DM/kg of gain) | Flight speed | During feedlot phase  (9x) | Angus (NSW popn) (n=49) | Finishing phase: 14-26 mo | Slope=-1.5 ± 0.81; P=0.07 | Cafe et al., 2011b |
|  | Flight speed (m/s) | 2x? (10- 12 mo) | Bos taurus (Angus, Charolais, Hybrid) (n=302) | In 70 d trial (10-12 mo) | Genetic: 0.40 ± 0.26  Phenotypic: 0.03 **NS** | Nkrumah et al., 2007 |
| RFI (kg/d) | Flight speed (m/s) | 2x? (10- 12 mo) | Bos taurus (Angus, Charolais, Hybrid) (n=302) | In 70 d trial (10-12 mo) | Genetic: -0.59 ± 0.45  Phenotypic = -0.07 **NS** | Nkrumah et al., 2007 |
|  | Flight speed | Finishing phase | Bos taurus (n=1141) | Finishing phase (8 mo to slaughter | Genetic:-0.07 ± 0.24  Phenotypic: -0.09 ± 0.04 | Rolfe et al., 2011 |

†Flight time is typically measured in s or s*100; flight distance in m. Scales for chute or other restraint tests and the docility test are shown with a character in bold indicating the excitable/nervous end of the scale.

*NS: author notes correlation as non-significant

Table A3. Table showing relationships between temperament traits and carcass traits and meat quality in beef cattle. (All measures taken post-mortem and all weights shown in kg).

| **Carcass traits and meat quality** | | | | | | |
| --- | --- | --- | --- | --- | --- | --- |
| **Trait** | **Temperament measure†** | **Age and frequency of temperament measure** | **Breed (sample size in parentheses)** | **Correlation*** | **Author(s)** |  |
| Carcass weight | Flight speed | 8-11x: ~12 mo | Bos indicus crossbreds  (n=96) | Regression coeff: 20.13; P<0.05 | Burrow and Dillon, 1997 |  |
|  |  |  | Bos indicus crossbreds  (n=119) | Regression coeff:-0.40; **NS** |  |  |
|  |  | During back-grounding at pasture  (5xin NSW)  (2x in WA) | Brahman (NSW popn) (n=163) | Slope: -9.9±2.9; P<0.01 | Cafe et al., 2011b |  |
|  |  |  | Brahman (WA popn) (n=143) | Slope: -9.7±3.9; P<0.05 |  |  |
|  |  |  | Angus (NSW popn) (n=49) | Slope: -27.0±14.5; P=0.07 |  |  |
|  |  | During feedlot phase (9x in NSW)  (2x in WA) | Brahman (NSW popn) (n=163) | Slope: -5.0± 2.9; P=0.09 |  |  |
|  |  |  | Brahman (WA popn) (n=143) | Slope: -6.4± 3.7; P=0.09 |  |  |
|  |  | 2x? (10- 12 mo) | Bos taurus (Angus, Charolais, Hybrid) (n=302) | Genetic: -0.54 ± 0.32; Phenotypic: -0.25; P<0.01 | Nkrumah et al., 2007 |  |
|  | Disposition score (response on exit from chute1-**6**: calm to excitable) | 3-5x during feedlot finishing | Bos taurus (Continental and British) (n= 21,528) | More excitable = lower hot carcass wt; P<0.001 | Reinhardt et al., 2009 |  |
|  | Crush score (1-**5**: calm to very nervous) | During feedlot phase (11x in NSW) | Brahman (NSW popn) (n=161) | Slope: -16.6±4.5; P<0.001 | Cafe et al., 2011b |  |
| Dressing % (carcass wt/liveweight*100) | Flight speed | 8-11x: ~12 mo | Bos indicus crossbreds  (n=96) | Regression coeff=-0.82; **NS** | Burrow and Dillon, 1997 |  |
|  |  |  | Bos indicus crossbreds  (n=119) | Regression coeff=-1.52; **NS** |  |  |
|  | Flight speed | Day 0 in feedlot (~29 mo) | Bos indicus crossbreds (n=120) | r = -0.20; P<0.05 | Petherick et al., 2002 |  |
| Fat thickness (cm) | Disposition score (response on exit from chute1-**6**: calm to excitable) | 3-5x during feedlot finishing | Bos taurus (Continental and British) (n= 21,528) | More excitable = lower fat thickness; P<0.001 | Reinhardt et al., 2009 |  |
| Carcass loin muscle area (cm^2^) | Flight speed (m/s) | 2x? (10- 12 mo) | Bos taurus (Angus, Charolais, Hybrid) (n=302) | Genetic:0.32 ± 0.25  Phenotypic: 0.14; P<0.05 | Nkrumah et al., 2007 |  |
|  | Disposition score (response on exit from chute1-6: calm to excitable) | 3-5x during feedlot finishing | Bos taurus (Continental and British)( n= 21,528) | More excitable = lower loin muscle area; P<0.001 | Reinhardt et al., 2009 |  |
| Carcass lean meat yield | Flight speed (m/s) | 2x? (10- 12 mo) | Bos taurus (Angus, Charolais, Hybrid) (n=302) | Genetic:0.33 ± 0.23  Phenotypic:0.30; P<0.001 | Nkrumah et al., 2007 |  |
| Carcass bruising | Vigour of movement in a race (1-7) | 12-42 mo | Bos inducus crossbreds (n=220) | Correlation: 0.06; **NS** | Fordyce et al., 1985 |  |
|  | Count of audible respiration when confined in a race |  |  | 0.05; **NS** |  |  |
|  | Temperament: sum of scores for fear responses to handling/confinement in a race |  |  | 0.08; **NS** |  |  |
|  | Flight speed | 8-11x: ~12 mo | Bos indicus crossbreds  (n=96) | Regression coeff=--0.01; **NS** | Burrow and Dillon, 1997 |  |
|  |  |  | Bos indicus crossbreds  (n=119) | Regression coeff=-0.03; **NS** |  |  |
| pH value post-slaughter | Flight speed | Day 0 in feedlot (~29 mo) | Bos indicus crossbreds (n=120) | r = -0.26; P<0.05: calm animals had higher pH | Petherick et al., 2002 |  |
|  |  | Day 21 in feedlot |  | r = -0.25; P<0.05 |  |  |
|  |  | Day 45 in feedlot |  | r = -0.25; P<0.01 |  |  |
|  | Temperament score (combined score from flight speed and pen score: high score= excitable) | 4x: start to end of feedlot finishing | Bos indicus cross and Bos taurus (Angus) (n=128) | Calm animals had higher pH post-mortem (P<0.05) | King et al., 2006 |  |
| Presence of μ–calpain marker | Flight speed | Periodically during routine handling | Brahman (173 from pop’n of 1,664) | Animals with 2 markers had higher FS than animals with fewer markers; P<0.05. No effects on other markers | Cafe- et al., 2010 |  |
| Warner Bratzler shear force (WBSF) (kg: high indicates meat toughness)  Warner Bratzler shear force (WBSF) (kg: high indicates meat toughness) | Flight time (s*100) | 1x? Post-weaning | Tropically adapted breeds(Brahman Belmont Red, Santa Gertrudis) (n=3594) | M longissimus thorasis +lumborum  Genetic: -0.48  Phenotypic:-0.01  M. semitendinosus:  Genetic: -0.17  Phenotypic: -0.05 | Reverter et al., 2003 |  |
|  | Flight time (s*100) | 8 mo  19 mo | Tropically adapted breeds(Brahman Belmont Red, Santa Gertrudis) (n=3594) | M longissimus thorasis +lumborum  Genetic: -0.42±0.02-0.03  Phenotypic: -0.02  M. semitendinosus:  Genetic: -0.17±0.02-0.03  Phenotypic: -0.05  M longissimus thorasis +lumborum  Genetic: -0.32±0.02-0.03  Phenotypic: -0.04  M. semitendinosus:  Genetic: -0.18±0.02-0.03  Phenotypic: -0.03 | Kadel et al., 2006  Kadel et al., 2006 (cont.) |  |
|  | Chute score (1-**15**: increasing movement in chute) | 8 mo  19 mo |  | M longissimus thorasis +lumborum  Genetic: 0.39±0.02-0.03  Phenotypic: 0.06  M. semitendinosus:  Genetic: 0.23±0.02-0.03  Phenotypic: 0.01  M longissimus thorasis +lumborum  Genetic: -0.37±0.02-0.03  Phenotypic: -0.01  M. semitendinosus:  Genetic: 0.11±0.02-0.03  Phenotypic: -0.05 |  |  |
|  | Temperament score (combined score from flight speed and pen score: high score= excitable) | 4x: start to end of feedlot finishing | Bos indicus cross and Bos taurus (Angus) (n=128) | SF at 21d aging:  Corr Coeff=0.21; P<0.10 | King et al., 2006 |  |
|  | Flight speed | Growing phase (at pasture) | Brahman (n=161, 137) and Angus (n=48, 16) popns | A number of measures for different hanging and aging procedures – generally show +ve and significant relationships with FS in growing and finishing phase | Cafe et al., 2011b |  |
|  | Exit velocity (Flight speed) | 7x from 5-8 mo until slaughter 14-16 mo | Commericial crossbred B*os taurus* steers (n=181) | Partial correlation: -0.182, P<0.05 | Hall et al., 2011 |  |
| Tenderness (consumer panel: 0-100:very tough – very tender) | Flight time (s*100) | 1x? Post-weaning | Tropically adapted breeds(Brahman Belmont Red, Santa Gertrudis) (n=3594) | Genetic: 0.37  Phenotypic:0.06 | Reverter et al., 2003 |  |
|  | Isolation score (1-**6**: response to isolation in a pen with handler: part of the docility test | 1 or 2x during fattening (15-18 mo) | Angus or Limousin crossbreds (n=144) | Calm animals had less tender meat; P<0.05 | Turner et al., 2011 |  |
| Marbling (traces-moderate) | Chute score: (1-**5**: calm to violently struggling) | 7x from 5-8 mo until slaughter (14-16 mo | Commericial crossbred B*os taurus* steers (n=181) | R^2^=0.087; P<0.05 | Hall et al., 2011 |  |
|  | Temperament score (combined score from flight speed and pen score: high score= excitable) | 4x: start to end of feedlot finishing | Bos indicus cross and Bos taurus (Angus) (n=128) | No association between temperament and marbling | King et al., 2006 |  |
|  | Flight speed (m/s) | 2x? (10- 12 mo) | Bos taurus (Angus, Charolais, Hybrid) (n=302) | Genetic: 0.10 ± 0.28  Phenotypic:-0.22; P<0.01 | Nkrumah et al., 2007 |  |
| Marbling score (small-modest) | Disposition score (response on exit from chute1-**6**: calm to excitable) | 3-5x during feedlot finishing | Bos taurus (Continental and British) (n= 21,528) | More excitable = lower marbling score; P<0.001 | Reinhardt et al., 2009 |  |
| Cooking Loss (%) from M longissimus thorasis +lumborum | Flight time (s*100) | 8 mo  19 mo | Tropically adapted breeds(Brahman Belmont Red, Santa Gertrudis) (n=3594) | Genetic: 0.22±0.02-0.03  Phenotypic: -0.00  Genetic: 0.30±0.02-0.03  Phenotypic: 0.00 | Kadel et al., 2006 |  |
|  | Chute score (1-**15**: increasing movement in chute) | 8 mo  19 mo |  | Genetic: -0.38±0.02-0.03  Phenotypic: 0.00  Genetic: -0.36±0.02-0.03  Phenotypic: 0.02 |  |  |

†Flight time is typically measured in s or s*100; flight distance in m. Scales for chute or other restraint tests and the docility test are shown with a character in bold indicating the excitable/nervous end of the scale.

*NS: author notes correlation as non-significant

Table A4. Table showing relationships between temperament traits and feeding behaviour for beef cattle.

| **Feeding behaviour** | | | | | | |
| --- | --- | --- | --- | --- | --- | --- |
| **Trait** | **Temperament measure†** | **Age and frequency of temperament measure** | **Breed (sample size in parentheses)** | **Age at which trait was measured** | **Correlation*** | **Author(s)** |
| Dry matter intake (kg of DM/d)  Dry matter intake (kg of DM/d)  (cont.) | Flight speed (m/s)  Flight speed (m/s) (cont.) | 2x? (10- 12 mo) | Bos taurus (Angus, Charolais, Hybrid) (n=302) | In 70 d trial (10-12 mo) | Genetic: -0.11± 0.26  Phenotypic: =-0.35 P<0.001 | Nkrumah et al., 2007 |
|  |  | Finishing phase | Bos taurus (n=1141) | Finishing phase (8 mo to slaughter | Genetic:-0.14 ± 0.25  Phenotypic: -0.22 ± 0.04 | Rolfe et al., 2011 |
|  |  | During back-grounding at pasture  (5xin NSW) | Brahman (NSW popn) (n=164) | In feedlot 12-14 to 17-19 mo | Slope=-0.37 ±0.15; P<0.05 | Cafe et al., 2011b |
|  |  | During feedlot phase (9x in NSW) | Brahman (NSW popn) (n=164) | In feedlot 12-14 to 17-19 mo | Slope=-0.26 ±0.14; P=0.07 |  |
| Dry matter intake (kg of DM/d) | Crush score (1-**5**: calm to very nervous) | During feedlot phase (9x in NSW) | Brahman (NSW popn) (n=164) | In feedlot 12-14 to 17-19 mo | Slope=-0.75 ±0.23; P<0.01 | Cafe et al., 2011b |
| Daily feeding duration (min/d) | Flight speed (m/s) | 2x? (10- 12 mo) | Bos taurus (Angus, Charolais, Hybrid) (n=302) | In 70 d trial (10-12 mo) | Genetic: 0.42 ± 0.26  Phenotypic: -0.03; **NS** | Nkrumah et al., 2007 |
| Daily feeding time (min/d) | Flight speed | During back-grounding at pasture  (5xin NSW)  (2x in WA) | Brahman (NSW popn) (n=164) | In feedlot 12-14 to 17-19 mo | Slope=-4.7 ±2.3; P<0.05 | Cafe et al., 2011b |
|  |  | During feedlot phase (9x in NSW)  (2x in WA) | Brahman (NSW popn) (n=164) | In feedlot 12-14 to 17-19 mo | Slope=-4.7 ±2.3; P<0.05 |  |
|  |  |  | Angus (NSW popn) (n=49) | In feedlot 14-16 to 19-21mo | Slope=-17.6 ±8.2; P<0.05 |  |
| Daily feeding head down time (min/d) | Flight speed (m/s) | 2x? (10- 12 mo) | Bos taurus (Angus, Charolais, Hybrid) (n=302) | In 70 d trial (10-12 mo) | Genetic -0.56 ± 0.38  Phenotypic -0.11 (P=0.10) | Nkrumah et al., 2007 |
| Daily feeding frequency (events/d) | Flight speed (m/s) | 2x? (10- 12 mo) | Bos taurus (Angus, Charolais, Hybrid) (n=302) | In 70 d trial (10-12 mo) | Genetic: -0.11 ± 0.30 **NS**  Phenotypic: 0.08; **NS** | Nkrumah et al., 2007 |
| Feeding session (No./d) | Crush score (1-**5**: calm to very nervous) | During back-grounding at pasture  (6xin NSW) | Angus (NSW popn) (n=49) | In feedlot 14-16 to 19-21mo | Slope=3.7 ±1.5; P<0.05 | Cafe et al., 2011b |
|  |  | During feedlot phase (11x in NSW) | Angus (NSW popn) (n=49) | In feedlot 14-16 to 19-21mo | Slope=4.9 ±2.3; P<0.05 |  |

†Flight time is typically measured in s or s*100; flight distance in m. Scales for chute or other restraint tests and the docility test are shown with a character in bold indicating the excitable/nervous end of the scale.

*NS: author notes correlation as non-significant

Table A5. Table showing relationships between temperament traits and reproductive traits for males and females in beef cattle

| **Reproduction** | | | | | | |
| --- | --- | --- | --- | --- | --- | --- |
| **Scrotal circumference** | | | | | | |
| **Trait** | **Temperament measure†** | **Age and frequency of temperament measure** | **Breed (sample size in parentheses)** | **Age at which trait was measured** | **Correlation*** | **Author(s)** |
| Scrotal circumference (mm) | Temperament: summed scores for fear responses to handling/confinement in a race – weaning (calm-excitable) | 24 mo | Bos indicus cross (n=485) | Weaning | Genetic: 0.72 ± 0.43 **NS**  Phenotypic: 0.09 | Fordyce et al., 1996 |
|  |  |  | (n=485) | 12 mo | Genetic: 1.04 ± 0.51 **NS** Phenotypic: 0.06 |  |
|  |  |  | (n=485) | 24 mo | Genetic: 0.54 ± 0.60 **NS** Phenotypic: 0.03 |  |
|  | Flight distance (minimum tolerated approach distance in a yard (m)) |  | Bos indicus cross (n=485) | Weaning | Genetic: -0.13 ± 0.31 **NS** Phenotypic: 0.02 |  |
|  |  |  | (n=312) | 12 mo | Genetic: 0.02 ± 0.34 **NS** Phenotypic: 0.03 |  |
|  |  |  | (n=485) | 24 mo | Genetic: 0.17 ± 0.32 **NS** Phenotypic: 0.13 |  |
|  | Flight speed | 3-6x: 6-18 mo | Bos indicus derived (Belmont Red)  (n=1871) | Weaning | Genetic: 0.13  Phenotypic: 0.11 | Burrow, 2001 |
|  |  |  |  | 12 mo | Genetic: 0.22  Phenotypic: 0.11 |  |
|  |  |  |  | 18 mo | Genetic: 0.11  Phenotypic: 0.07 |  |
|  | Flight speed | Yearling | Nellore (n=7402) | 510 d | Genetic: -0.11 ± 0.07  Phenotypic: -0.07 ± 0.02 | Sant’Anna et al., 2012 |
|  | Temperament score – response to handling and human presence (1-**4**: good-poor) | 1x: yearling | Bos indicus (Nellore)  (n=18,891) | 500 days  (16-17 mo) | Genetic: -0.07± 0.07  Phenotypic: -0.02 ±0.03 | Barrozo et al., 2012 |
| Expression of oestrus (count) | Flight speed | Mature | Bos indicus-derived (Belmont Red)  (n=120) | Mature | Docile>temperamental  (P<0.05) | Burrow et al., 1988 |
| Reproductive tract functioning; conception rate |  |  | Bos indicus-derived (Belmont Red)  (n=120) |  | No difference between docile and temperamental |  |
| Age at puberty | Docility score (6.5-17: aggressive to docile) | 1x 10-14 mo | Limousin (n=2781) | ~14 mo | Genetic: -0.32 ± 0.14; P<0.05 | Phocas et al., 2006 |
| Age at first calving | Temperament score – response to handling and human presence (1-**4**: good-poor) | 1x: yearling | Bos indicus (Nellore)  (n=18,891) | ~35 mo | Genetic: -0.06 ± 0.19  Phenotypic: 0.05 ± 0.05 | Barrozo et al., 2012 |
| Pregnancy status after 3 matings (yes/no) | Flight speed | Measures 3-6x; 6-18 mo | Bos indicus derived (Belmont Red)  (n=913) | Mature | Genetic: 0.00  Phenotypic: 0.00 | Burrow, 2001 |
| Days to calving (following mating) |  |  |  |  | Genetic: 0.15  Phenotypic: 0.01 |  |
| Fertility (calving after AI) | Docility score (6.5-17: aggressive to docile) | 1x 10-14 mo | Limousin (n=2781) | ~24 mo | Genetic: 0.55 ± 0.22  P<0.05 | Phocas et al., 2006 |
| Calving ease |  |  |  |  | Genetic: 0.13 ± 0.14 **NS** |  |
| Pelvic opening |  |  |  |  | Genetic: 0.23 ± 0.13 **NS** |  |
| Maternal behavior |  |  |  |  | Genetic: 0.17 ± 0.11 **NS** |  |
| Milk yield |  |  |  |  | Genetic: 0.07 ± 0.10 **NS** |  |

†Flight time is typically measured in s or s*100; flight distance in m. Scales for chute or other restraint tests and the docility test are shown with a character in bold indicating the excitable/nervous end of the scale.

*NS: author notes correlation as non-significant

Table A6. Table showing relationships between temperament traits and measures of stress physiology in beef cattle

| **Stress physiology** | | | | | | |
| --- | --- | --- | --- | --- | --- | --- |
| **Trait** | **Temperament measure†** | **Age and frequency of temperament measure** | **Breed (sample size in parentheses)** | **Age at which trait was measured** | **Correlation*** | **Author(s)** |
| Cortisol levels (nmol/L) | ‘Temperament’: (calm and nervous: combination of flight time, crush score and willingness to move past humans to food) | 2x: weaning and 21d post-weaning | Angus x Hereford and Hereford (n=24, selected from a group of 209) | Before weaning (before 7-9 mo) | Calm animals had lower plasma cortisol levels (P<0.01) | Fell et al., 1999 |
|  |  |  |  | After weaning (7-9 mo) | Calm animals had lower plasma cortisol levels (P<0.01) |  |
|  |  |  |  | On entry to feedlot (13-15 mo) | Calm animals had lower plasma cortisol levels (P<0.05) |  |
| Rectal temperature (°C) | Flight speed | 3-6x: 6-18 mo | Bos indicus derived (Belmont Red)  (n=1871) | 4-7 counts | Genetic: -0.06  Phenotypic: -0.24 | Burrow, 2001 |
|  | Flight time | 1-9x 8-18 mo | Bos taurus derived and  Bos indicus derived (n~2555) | 3x post-weaning | Phenotypic:  Zebu: -0.18 ±0.06-0.07; P<0.01  British -0.15 ±0.06-0.07; P<0.01 | Prayaga, 2003 |
|  | Flight time | 1-9x 8-18 mo | Bos taurus derived and  Bos indicus derived (n~2555) | 3x post-weaning | Genetic:-0.37 ± 0.01-0.18 **P<0.05**  Phenotypic -0.11 ± 0.01-0.03 **P<0.05** | Prayaga and Henshall, 2005 |
| Cortisol (ng/mL)  Cortisol (ng/mL) (cont.) | Chute score (1-**5**: quiet to excited)  Flight speed (m/s)  Pen score (1-**5**: quiet to excited) | 3x: Day 0, 60 and 120 (12-15 mo of age) | Brahman bulls (n=66) | 12 months | Day 0: r=0.09; NS  Day 0: r=0.26; P<0.05  Day 120: r=0.44; P<0.001  Day 0: r=0.29; P<0.05  Day 120: r=0.25; P<0.05 | Curley et al., 2006a |
|  | Flight speed (m/s) | 1x: on entry to feedlot | Cross-bred steers (n=150) | 4x: Pre- and post-transportation to feedlot, during finishing (d70) and at slaughter (SLTR) | Pre- and post transport: Temperamental animals higher in cortisol (P<0.05)  D70: Temperamental animals higher in cortisol (P<0.01)  Reduced effect at slaughter (P=0.06) | Curley et al., 2006b |
|  | Temperament score (combined score from flight speed and pen score: high score= excitable) | 4x: start to end of feedlot finishing | Bos indicus cross and Bos taurus (Angus) (n=128) | Same as temperament: 4x: start-end feedlot finishing | Excitable animals had higher cortisol (P<0.05) | King et al., 2006 |
|  | Temperament score (exit velocity and pen score)(low-high=calm-excitable) | 1 x pre-weaning | Brahman bulls: 7 calm, 8 temperamental from a pool of 60 | 10 months | Cortisol increased due to transportation in calm bulls only (P<0.01) | Burdick et al., 2011 |
| Epinephrine (pg/mL) | Flight speed (m/s) | 1x: on entry to feedlot | Cross-bred steers (n=150) | 4x: Pre- and post-transportation to feedlot, during finishing (d70) and at slaughter (SLTR) | Temperamental animals higher levels pre-and post transport and d70 (P<0.03) but not at slaughter | Curley et al., 2006b |
|  | Temperament score (exit velocity and pen score)(low-high=calm-excitable) | 1 x pre-weaning | Brahman bulls: 7 calm, 8 temperamental from a pool of 60 | 10 months | Epinephrine decreased due to transportation in temperamental bulls only (P=0.07) | Burdick et al., 2011 |
|  | Flight speed/exit velocity (m/s) | 2 x: 21-24 d after birth and weaning | Brahman (n=116) | 6x: 0-24 days of life | No relationship with neonatal temperament | Burdick et al., 2009 |
| Glucose (ng/dl) and cortisol ((ng/ml) after transportation | Temperament: exit velocity and pen score | 1x: Pre-weaning | Brahman (n=16 from a pool of 59) | 11-12 mo | Excitable animals had higher glucose and cortisol levels after transportation (P<0.01) | Hulbert et al., 2011 |

†Flight time is typically measured in s or s*100; flight distance in m. Scales for chute or other restraint tests and the docility test are shown with a character in bold indicating the excitable/nervous end of the scale.

*NS: author notes correlation as non-significant

Table A7. Table showing relationships between temper ament traits and measures of health and body condition in beef cattle

| **Health** | | | | | | |
| --- | --- | --- | --- | --- | --- | --- |
| **Trait** | **Temperament measure†** | **Age and frequency of temperament measure** | **Breed (sample size in parentheses)** | **Age at which trait was measured** | **Correlation*** | **Author(s)** |
| Hospitalisations (% of group hospitalized) | ‘Temperament’: (calm and nervous: combination of flight time, crush score and willingness to move past humans to food) | 2x: weaning and 21d post-weaning | Angus x Hereford and Hereford (n=24, selected from a group of 209) | Finishing (13-15 mo to slaughter) | Calm animals had fewer hospitalisations (P<0.05); Nervous group: 42% vs. 0%: calm group | Fell et al., 1999 |
| Body condition score (1-9: emaciated to over-fat) | Flight speed (m/s) | Day 0 in feedlot feedlot (~29 mo) | Bos indicus crossbreds (n=120) | Day 0 in feedlot feedlot (~29 mo) | r = -0.26; P<0.01 | Petherick et al., 2002 |
|  |  | Day 0 in feedlot |  | Day 21 in feedlot | r = -0.25; P<0.01 |  |
|  |  | Day 0 in feedlot |  | Day 70 in feedlot | r = -0.33; P<0.01 |  |
|  |  | Day 0 in feedlot |  | Day 101 in feedlot | r = -0.23; P<0.05 |  |
|  |  | Day 21 in feedlot |  | Day 21 in feedlot | r = -0.29; P<0.01 |  |
|  |  | Day 70 in feedlot |  | Day 70 in feedlot | r = -0.24; P<0.01 |  |
|  |  | Day 101 in feedlot |  | Day 101 in feedlot | r = -0.38; P<0.01 |  |
| Treatment for respiratory disease | Disposition score (response on exit from chute1-**6**: calm to excitable) | 3-5x during feedlot finishing | Bos taurus (Continental and British) (n= 21,528) | Feedlot finishing | No effect of temperament | Reinhardt et al., 2009 |
| Presence of lung lesions post-mortem | Disposition score (response on exit from chute1-**6**: calm to excitable) | 3-5x during feedlot finishing | Bos taurus (Continental and British) (n= 21,528) | Feedlot finishing | No effect of temperament |  |
| Mortality | Disposition score (response on exit from chute1-**6**: calm to excitable) | 3-5x during feedlot finishing | Bos taurus (Continental and British) (n= 21,528) | Feedlot finishing | Increasing mortality with poor temperament in steers but not heifers (P<0.01) |  |
| Immunoglobulins (IgA, IgM, IgG, IgG_1_, IgG_2_ (mg.mL) | Flight speed/exit velocity (m/s) | 2 x: 21-24 d after birth and weaning | Brahman (n=116) | 6x: 0-24 days of life | No relationship with neonatal temperament | Burdick et al., 2009 |
| IgM (mg/100mL) | ‘Temperament’: (calm and nervous: combination of flight time, crush score and willingness to move past humans to food) | 2x: weaning and 21d post-weaning | Angus x Hereford and Hereford (n=24, selected from a group of 209) | Feedlot finishing: 13-15 mo to slaughter | Calm animals lower IgM than nervous animals (P<0.05) | Fell et al., 1999 |
| IgA (mg/100 mL) |  |  |  |  | No difference between calm and nervous groups |  |
| Leucocyte counts |  |  |  |  | No difference between calm and nervous groups |  |
| Lymphocyte proliferation (stimulation index) |  |  |  |  | No difference between calm and nervous groups |  |
| Interferon production (activity) |  |  |  |  | No difference between calm and nervous groups |  |
| Natural Killer Cell activity (% target lysis) |  |  |  |  | No difference between calm and nervous groups |  |
| Tick count | Temperament: summed scores for fear responses to handling/confinement in a race (calm-excitable) | Weaning | (n=485) | 24 mo | Phenotypic: -0.14 **NS** | Fordyce et al., 1996 |
|  |  | 12 mo | (n=485) |  | Phenotypic: -0.07 **NS** |  |
|  |  | 24 mo | (n=485) |  | Phenotypic: -0.10 **NS** |  |
|  | Flight distance (minimum tolerated approach distance in a yard (m)) | Weaning | Bos indicus cross (n=485) |  | Phenotypic: -0.06 **NS** |  |
|  |  | 12 mo | (n=312) |  | Phenotypic: -0.09 **NS** |  |
|  |  | 24 mo | (n=485) |  | Phenotypic: -0.06 **NS** |  |
|  | Flight speed | 3-6x: 6-18 mo | Bos indicus derived (Belmont Red)  (n=1871) | 1-7 counts: 6-18 mo | Genetic: -0.01  Phenotypic: 0.05 | Burrow, 2001 |
|  | Flight time | 1-9x 8-18 mo | Bos taurus derived and  Bos indicus derived (n~2555) | 1-4 counts of ticks | Phenotypic:  Zebu: -0.05 ±0.06-0.07 **NS**  British: 0.01 ±0.06-0.07 **NS** | Prayaga, 2003 |
|  | Flight time | 1-9x 8-18 mo | Bos taurus derived and  Bos indicus derived (n~2555) | 1-4 counts of ticks | Genetic:-0.17 ± 0.01-0.18  Phenotypic 0.01 ± 0.01-0.03 **NS** | Prayaga and Henshall, 2005 |
| Fly count | Flight speed | Measures 3-6x | Bos indicus derived (Belmont Red)  (n=1871) | 4-8 counts: 6-18 mo | Genetic: 0.00  Phenotypic: 0.00 | Burrow, 2001 |
| Worm egg count | Flight speed | Measures 3-6x | Bos indicus derived (Belmont Red)  (n=1871) | 3-7 egg counts: 6-18 mo | Genetic: 0.00  Phenotypic: -0.02 |  |
|  | Flight time | 1-9x 8-18 mo | Bos taurus derived and  Bos indicus derived (n~2555) | 1-6 egg counts | Phenotypic:  Zebu: 0.07 ±0.06-0.077 **NS**  British: 0.16 ±0.06-0.07 | Prayaga, 2003 |
|  | Flight time | 1-9x 8-18 mo | Bos Taurus derived and  Bos indicus derived (n~2555) | 1-6x egg counts | Genetic: -0.15 ± 0.01-0.18  Phenotypic 0.02 ± 0.01-0.03 **NS** | Prayaga and Henshall, 2005 |
| Shedding of E. Coli O157:H7 (presence/absence in repeated tests) | Flight speed/exit velocity | Pre- and post-arrival at the feedlot | Commercial cattle (n=150) | 4x: during feedlot period and at harvesting | Calm animals shed more E. Coli (shedding indicative of stress) (P<0.05) | Schuehle Pfeiffer et al., 2009 |
| Neutrophil function after transportation | Temperament: exit velocity and pen score | 1x: Pre-weaning | Brahman (n=16 from a pool of 59) | 11-12 mo | After transportation, neutrophils from calm bulls more likely to resist microbial invasionthan those from temperamental bulls | Hulbert et al., 2011 |

*NS: author notes correlation as non-significant

†Flight time is typically measured in m*100; flight distance in m. Scales for chute or other restraint tests and the docility test are shown with a character in bold indicating the excitable/nervous end of the scale.

Table B1Table showing relationships of temperament traits to other traits in dairy cattle. Measures always taken on adult cows, generally at first lactation.

| **DAIRY CATTLE** | | | | | | | | |
| --- | --- | --- | --- | --- | --- | --- | --- | --- |
| **Trait** | **Temperament measure†** | **Age and frequency of temperament measure** | | **Breed (sample size in parentheses)** | | **Score for correlated measure** | **Correlation*** | **Author(s)** |
| **Health and survival** | | | | | | | | |
| Mastitis resistance | Temperament (**1**-50: excitable to docile) | 1x >=24 mo | Holstein  (n=9546) | | Score from 1-50: susceptible to resistant | | Genetic: 0.46±0.18  Phenotypic: 0.13±0.11 | Lawstuen et al., 1988 |
| General health |  |  |  |  | Score from 1-50: sickly to vigorous | | Genetic: 0.37±0.22  Phenotypic: 0.11±0.10 |  |
| Udder edema |  |  |  |  | Score from 1-50: chronic to none | | Genetic:0.20±0.14  Phenotypic: 0.10±0.09 |  |
| Somatic cell count | Willingness to approach (% of cows observed that approached to 1m) | Once | Holstein  (41 farms) | | Counts (‘000) | | Phenotypic: r=-0.31 P=0.05 | Fulwider et al., 2007 |
| Somatic cell score | Temperament (scored **1**-5 very nervous to very calm) | 1x adult (1^st^ lactation) | Holstein  EBVs for 4109 bulls | | (counts ‘000) | | Bull EBV correlation: 0.190; P<0.01 | Sewalem et al., 2011 |
| Survival to 2^nd^ lactation | Adaptability (to herd after calving) (**1**-9)  Milking temperament  (**1**-9)  Adaptability (to herd after calving) (**1**-9)  Milking temperament  (**1**-9)  Adaptability (to herd after calving) (**1**-9)  Milking temperament  (**1**-9) | 1x adult | Holstein (n=59,623); 1116 sires  Jersey (n=45,396);  773 sires  Ayrshire (n=6599);  210 sires | | 0-1 score | | r_g_=0.487±0.06  r_p_=0.081±0.005  r_g_=0.375 ±0.06  r_p_=0.081 ±0.005  r_g_=0.439±0.06  r_p_=0.080±0.005  r_g_=0.383 ±0.06  r_p_=0.089 ±0.005  r_g_=0.142±0.15  r_p_=0.109±0.02  r_g_=0.200 ±0.15  r_p_=0.121 ±0.02 | Cue et al., 1996  Cue et al., 1996 |
| Survival heifer to 3^rd^ lactation | Adaptability (to herd after calving)  (1-9)  Milking temperament  (1-9)  Adaptability (to herd after calving)  (1-9)  Milking temperament  (1-9)  Adaptability (to herd after calving)  (1-9)  Milking temperament  (1-9) | 1 x adult | Holstein (n=59,623); 1116 sires  Jersey (n=45,396);  773 sires  Ayrshire (n=6599);  210 sires | | 0-1 score | | r_g_=0.383±0.005  r_p_=0.080±0.005  r_g_=0.302 ±0.06  r_p_=0.082 ±0.005  r_g_=0.401±0.005  r_p_=0.093±0.005  r_g_=0.347 ±0.06  r_p_=0.097 ±0.005  r_g_=-0.013±0.15  r_p_=0.112±0.02  r_g_=0.146 ±0.15  r_p_=0.126 ±0.02 |  |
| Survival | Temperament (A-E: placid-nervous) | 1x adults | Holstein Friesian  (n~200,000 ) | | 0-1 score at each parity | | Genetic: 0.64 ± 0.15 | Haile-Mariam et al., 2004 |
| Functional longevity | Temperament (scored **1**-5 very nervous to very calm) | 1x adult (1^st^ lactation) | Holstein: (n=1,728,289)  Jersey: (n=39,618)  Ayrshire: (n=54391) | | Length of productive life from 1^st^ calving to cull/death (d) | | Very nervous cows more likely to be culled: Holstein 26%, Jersey 46%, Ayrshire 23% | Sewalem et al., 2010 |
| **Production** | | | | | | | | |
| Milking speed | Temperament (**1**-50: excitable to docile) | 1x >=24 mo | Holstein  (n=9546) | | Score from 1-50: slow-fast | | Genetic:0.36±0.11  Phenotypic: 0.14±0.11 | Lawstuen et al., 1988 |
| Milking speed | Temperament (scored **1**-5 very nervous to very calm) | 1x adult (1^st^ lactation) | Holstein: (n=1,728,289)  Jersey: (n=39,618)  Ayrshire: (n=54391) | | Score **1**-5: very slow to very fast | | Genetic: 0.25  Phenotypic: 0.15; P<0.01 | Sewalem et al., 2011 |
| Yield | Temperament (**1**-50: excitable to docile) | 1x >=24 mo | Holstein  (n=9546) | | Fat corrected milk (kg) | | Genetic: 0.19±0.11  Phenotypic: 0.12±0.11 | Lawstuen et al., 1988 |
| Yield | Human approach (time spent within 3m of human) | Adult lactating  Adult lactating  (cont.) | Holstein Friesian (cows from 31 farms)  Holstein Friesian (cows from 31 farms) | | Milk yield (l/cow/year) | | Corr Coeff = 0.46; P<0.05 | Breuer et al., 2000  Breuer et al., 2000 (cont.) |
| Yield- protein |  |  |  |  | Protein (kg/cow/year) | | Corr Coeff = 0.49; P<0.01 |  |
| Yield - fat |  |  |  |  | Milk fat  (kg/cow/year) | | Corr Coeff = 0.43; P<0.05 |  |
| Yield | No. of flinch and step response when cups attached |  |  |  | Milk yield (l/cow/year) | | Corr Coeff = -0.37; P<0.05 |  |
| Yield- protein |  |  |  |  | Protein (kg/cow/year) | | Corr Coeff = -0.39; P<0.05 |  |
| Yield - fat |  |  |  |  | Milk fat  (kg/cow/year) | | Corr Coeff = -0.25; NS |  |
| **Reproduction** | | | | | | | | |
| Reproductive performance | Temperament (**1**-50: excitable to docile) | 1x >=24 mo | | Holstein  (n=9546) | | Score from 1-50: low-high | Genetic:0.30±0.34 **NS**  Phenotypic: 0.08±0.08 **NS** | Lawstuen et al., 1988 |
| Calving ease |  |  |  |  |  | Score from **1**-50: hard-easy | Genetic:0.48±0.18  Phenotypic: 0.12±0.10 |  |
| Calving ease | Temperament (1-5 very nervous to very calm) | 1x adult (1^st^ lactation) | | EBVs for 4109 bulls | | Score 1-4: unassisted-surgery | Bull EBV correlation:  -0.11; P<0.01 | Sewalem et al., 2011 |
| Days open |  |  |  |  |  | Calving to conception (d) | Bull EBV correlation:  -0.028; P<0.01 |  |
| Calving interval | Milking temperament (**1**-9: nervous-quiet) | 1x adult (1^st^ lactation) | | Holstein  (n=44,672) | | (1^st^-2^nd^ calving (d) | 0.24±0.14 | Pryce et al., 2000 |
| Calving interval | Temperament (A-E: placid-nervous) | 1x adult | | Holstein Friesian  (n~220,000) | | 1^st^-2^nd^ calving (d) | Genetic: 0.05 ± 0.15 | Haile-Mariam et al., 2004 |
| First service non-return rate | Temperament (A-E: placid-nervous) | 1x adult | | Holstein Friesian  (n~74,000) | | (0/1) | Genetic: -0.38 ± 0.15 |  |
| Age at first service | Temperament (scored **1**-5 very nervous to very calm) | 1x adult (1^st^ lactation) | | Holstein  EBVs for 4109 bulls | | Mo | Bull EBV correlation:  0.026; P<0.1 | Sewalem et al., 2011 |

*NS: author notes correlation as non-significant

†Temperament is typically scored by producers/farmers. Flight time is typically measured in s or s*100; flight distance in m. Scales for chute or other restraint tests and the docility test are shown with a character in bold indicating the excitable/nervous end of the scale.

All weights are in kg.
